# Radiobiological effects of wound fluid on breast cancer cell lines and human-derived tumor spheroids in 2D and microfluidic culture

**DOI:** 10.1038/s41598-022-11023-z

**Published:** 2022-05-10

**Authors:** Shabnam Jeibouei, Ali Hojat, Ebrahim Mostafavi, Amir Reza Aref, Alireza Kalbasi, Vahid Niazi, Mohammad Ajoudanian, Farzaneh Mohammadi, Fariba Saadati, Seyed Mohammadreza Javadi, Forough Shams, Maryam Moghaddam, Farshid Karami, Kazem Sharifi, Farid Moradian, Mohammad Esmaeil Akbari, Hakimeh Zali

**Affiliations:** 1 Cancer Research Center, Shahid Beheshti University of Medical Sciences, Tehran, Iran; 2 Department of Medical Biotechnology, School of Advanced Technologies in Medicine, Shahid Beheshti University of Medical Sciences, Tehran, Iran; 3 Stanford Cardiovascular Institute, Stanford University School of Medicine, Stanford, CA USA; 4 Department of Medicine, Stanford University School of Medicine, Stanford, CA USA; 5 Xsphera Biosciences Inc., 6 Tide street, Boston, USA; 6 Belfer Center for Applied Cancer Science, Dana-Farber Cancer Institute, Boston, MA USA; 7 Brigham and Women’s Hospital, Harvard Medical School, Boston, MA USA; 8 Department of Tissue Engineering and Applied Cell Sciences, School of Advanced Technologies in Medicine, Shahid Beheshti University of Medical Sciences, Tehran, Iran; 9 Department of Biology, Central Tehran Branch, Islamic Azad University, Tehran, Iran; 10 ZIK Plasmatis, Leibniz Institute for Plasma Science and Technology (INP), Greifswald, Germany; 11 Department of Surgery, School of Medicine, Besat Hospital, Hamadan University of Medical Sciences, Hamadan, Iran; 12 Department of Molecular and Cell Biology, Faculty of Life Sciences and Biotechnology, Shahid Beheshti University, Tehran, Iran; 13 Shohadaye Tajrish Hospital, Shahid Beheshti University of Medical Sciences, Tehran, Iran

**Keywords:** Biological techniques, Biophysics, Cell biology, Computational biology and bioinformatics, Medical research, Oncology, Risk factors, Cancer, Breast cancer

## Abstract

Intraoperative radiotherapy (IORT) could abrogate cancer recurrences, but the underlying mechanisms are unclear. To clarify the effects of IORT-induced wound fluid on tumor progression, we treated breast cancer cell lines and human-derived tumor spheroids in 2D and microfluidic cell culture systems, respectively. The viability, migration, and invasion of the cells under treatment of IORT-induced wound fluid (WF-RT) and the cells under surgery-induced wound fluid (WF) were compared. Our findings showed that cell viability was increased in spheroids under both WF treatments, whereas viability of the cell lines depended on the type of cells and incubation times. Both WFs significantly increased sub-G1 and arrested the cells in G0/G1 phases associated with increased P16 and P21 expression levels. The expression level of Caspase 3 in both cell culture systems and for both WF-treated groups was significantly increased. Furthermore, our results revealed that although the migration was increased in both systems of WF-treated cells compared to cell culture media-treated cells, E-cadherin expression was significantly increased only in the WF-RT group. In conclusion, WF-RT could not effectively inhibit tumor progression in an ex vivo tumor-on-chip model. Moreover, our data suggest that a microfluidic system could be a suitable 3D system to mimic in vivo tumor conditions than 2D cell culture.

## Introduction

Breast cancer is the most common malignancy in women worldwide^1,2^, and intraoperative radiotherapy (IORT) has been established as an appropriate type of treatment in these patients^3^. IORT directly delivers a single-radiation in a high-dose fraction to the tumor bed after the tumor's excision during Breast-Conserving Surgery (BCS)^4^. Epidemiological studies on IORT-treated breast cancer patients show lower local and distant recurrence rates than the non-IORT-treated group^5^. A recent study, which compared the outcome following application of full dose electron beam IORT in 216 early breast cancer patients and 323 external-beam-irradiated cases, revealed that IORT resulted in a lower rate of local recurrence, while external beam radiotherapy resulted in a higher rate of death and systemic recurrence^6^. Furthermore, a 4-year survival study revealed a significantly lower rate of metastases and ipsilateral breast tumor recurrence in Intraoperative Electron Radiotherapy (IOERT)-treated versus conventional radiotherapy-treated patients^7^. Published data suggest that, compared with whole breast radiation therapy, treatment with IORT not only reduces the treatment time^8^ but also appears to alter the microenvironment of the irradiated tumor bed. The surgical procedure initiates a wound healing response that makes the tumor bed microenvironment favorable to the growth of the remaining tumor cells^9,10^. These can proliferate and cause the development of loco-regional and distant recurrences resulting from the hypoxic microenvironment^11,12^. Considerable evidence suggests that radiation administration substantially impacts the surgical wound fluid composition and biological activity of fluid^10,13^. Our previous proteomic and transcriptomic studies on tumor bed tissue showed that IOERT (both radical and boost dose) alters various molecular pathways^14^. In this study, data analysis showed that both irradiation doses resulted in the upregulation of signaling pathways, including those involving TNF, NF-kappa B, PI3K-AKT, FoxO, and HIF-1. We also found upregulation of apoptosis, Toll-like receptor, B cell receptor, and metabolic pathways, the engagement of which is known to have both local and systemic effects, respectively^14^. Additional studies have suggested that wound fluid (WF) from irradiated tumor beds might be beneficial in reducing *loco* recurrence due to abrogation of proliferation, migration, and invasion of cancer cells^15–17^. The specific effects of surgery and radiation on the tumor bed are still mainly unexplored, and the function of the tumor microenvironment in response to radiation is not clearly understood. A tumor is a diverse microsystem that, in addition to cancer cells, includes extracellular matrix (ECM), immune and stromal cells interacting with cancer cells, thus impacting the disease itself^18^. There is increasing progress in developing microfluidic devices with multiple functions and various biological applications^19^. As a promising three-dimensional (3D) platform, the microfluidic system can aid in studying tumor in vivo processes. This system simulates the tumor microenvironment for patient-derived tumor spheroids^20^. 3D culture of human-derived tumor spheroids in microfluidic chips proposes this system as an appropriate approach to evaluate ex-vivo responses^21,22^. It is broadly affirmed that cell proliferation is different when cultured in a two-dimensional (2D) cell culture system than in 3D conditions^23^. In addition, recent data linked with mathematical models also explained how the stiffness of the substrate could control the growth of cancer cells ^24,25^. Tissue stiffness is a crucial factor of the ECM that contributing to epithelial-mesenchymal transition (EMT). In breast cancer cells, increasing stiffness of the surrounding ECM stimulates EMT^26^. To our knowledge, no study has evaluated the effects of irradiated WF on human-derived tumor cells in a microfluidic system. Most of the recent investigations evaluated the effects of surgical WF on cancer cells and compared them with the effect of WF-RT (irradiation-induced WF) on breast cancer cells in 2D systems.

**Figure 1:**
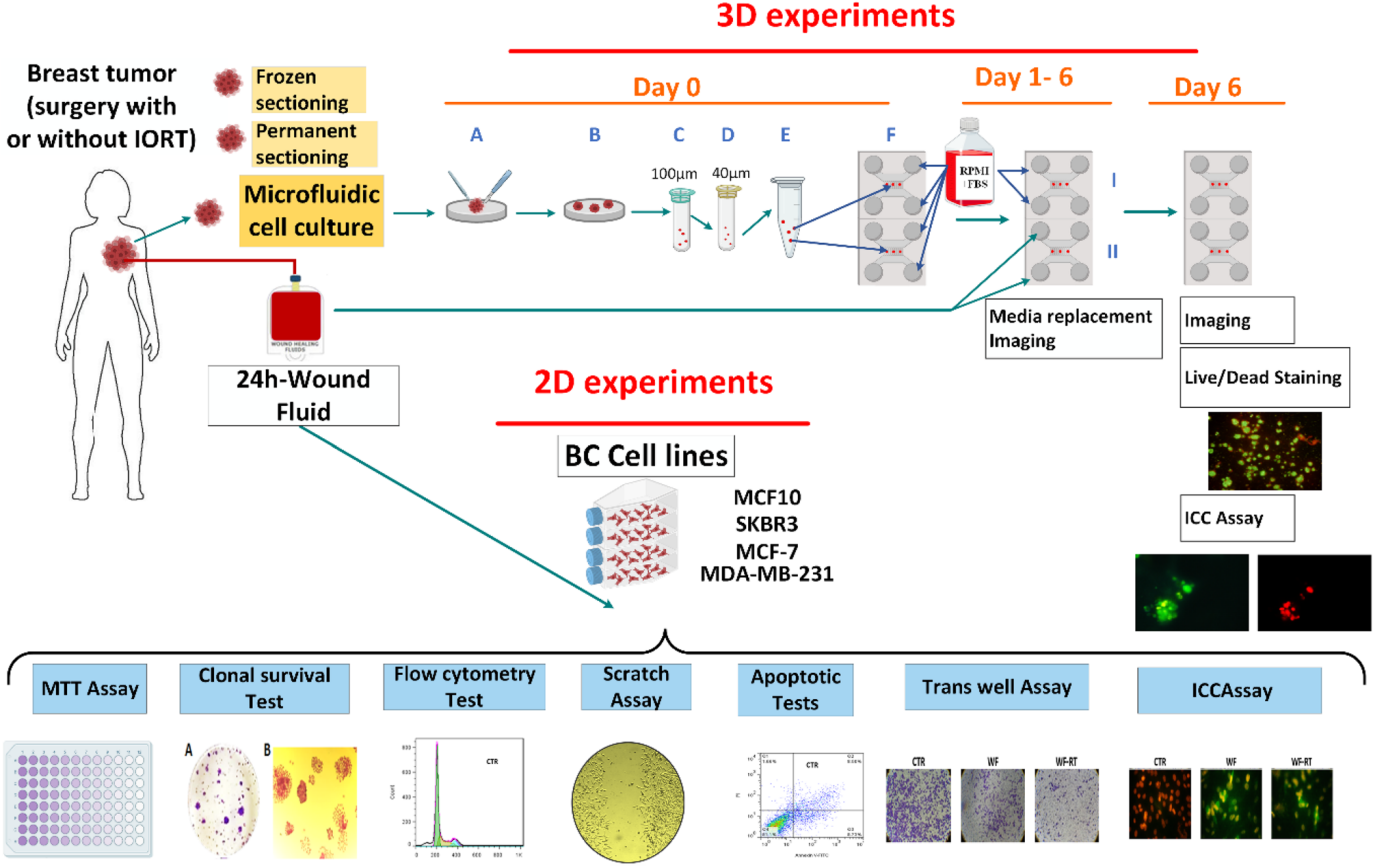
The graphical abstract of the study. Patients were classified into two groups: the Control group (only surgery) and the Test group (surgery + IORT). 3D experiments: On day 0, (**A**, **B**) Mechanically and enzymatically dissociation of the tumor specimens, respectively. (**C**, **D**) Filtration of the dissociated specimens using 100 µm and 40 µm cell strainers, respectively. (**E**) Embedding the spheroids into prepared collagen gel solution. (**F**) Filling the gel (collagen + spheroids) into the central channels and RPMI + FBS into media channels of the microfluidic devices. On days 1–6, I: Control device whose side channels are loading up with RPMI + FBS. II: Test device whose media channels are loading up with 24 h-wound fluid (WF/WF-RT). Optical imaging and media replacement were accomplished from day 0 to day 6 (RPMI + FBS for control devices and 24 h-wound fluid for test devices). On day 6, Live/Dead staining, immunocytochemistry, and fluorescent imaging. 2D experiments: assays on breast cancer cell lines under 24 h-WF/WF-RT treatment. The figure was created using Biorender (https://biorender.com).

In this study, we measured the viability of 3D-human-derived tumor spheroids in microfluidic culture systems as a model of tumor cells in their natural environment containing ECM, immune cells, and stromal cells under treatment with surgical WF, with and without IORT. We hope to illustrate better cellular behavior in 3D tumor spheroids than in 2D cell culture. Also, Caspase 3 and E-cadherin's expression levels in spheroids derived from IORT-treated and untreated human breast tumors were assessed to understand the rate of apoptosis and migration. Furthermore, we cultured four breast cancer cell lines in a 2D cell culture system and evaluated the factors related to viability and proliferation, apoptosis, migration, and invasion of cells under the treatment of WF-RT. Then, we went ahead and compared the results from the 3D microfluidic culture of human-derived spheroids with 2D monolayer cell lines. We conclude that this study will provide a better understanding of the role of ex vivo 3D modeling of human tumors and the effect of WF/WF-RT in several processes in 2D and 3D culture systems. The graphical abstract of the study is represented in Fig. 1.

## Results

### WF induce viability and proliferation in 2D culture of breast cancer cell lines

**Figure 2:**
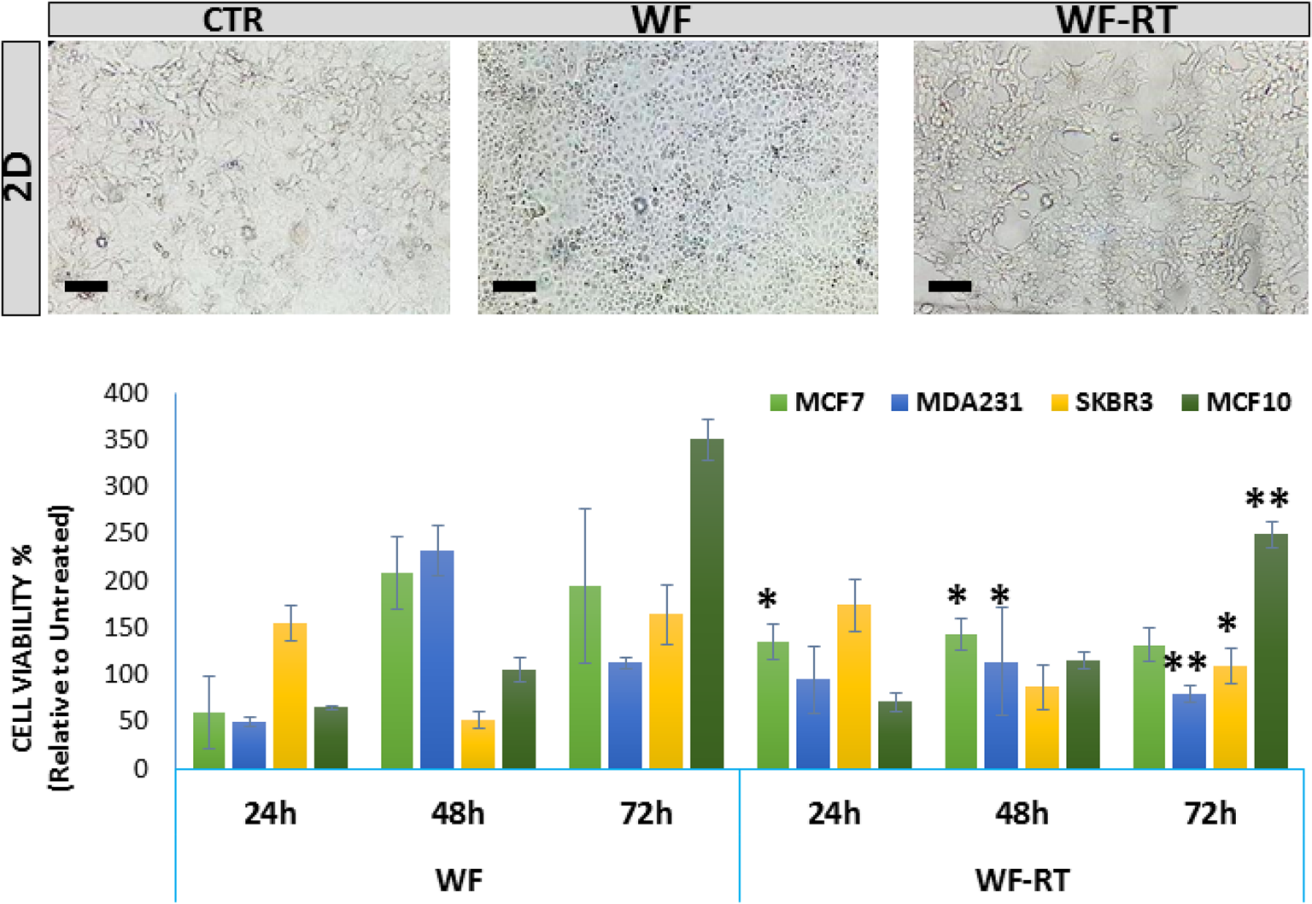
The microscope images and cell viability were obtained by MTT assay for BC cell lines under IORT and non-IORT wound fluid treatment. Breast cancer cells were treated with 10% concentrations of WF/WF-RT for 24, 48, and 72 h, and the inverted light microscopic images were obtained from MCF7 cells treated with WFs and DMEM after 24 h. Data were presented as mean ± SD (n = 12). All treated cells were assessed with their control, including those treated with DMEM without any WFs. The control group is not shown in the graph (cell viability = 100%). WF-RT-treated cells were compared with WF-treated cells for each cell line, and stars represent a significant value. *P < 0.05, and **P < 0.01. *CTR* control (DMEM + 10% FBS), *WF* wound fluid, *WF-RT* IORT-affected wound fluid.

To investigate the effects of WF/WF-RT on cell viability, three breast cancer cell lines with different histopathological characteristics, MCF-7, MDA-MB-231, SKBR-3, and a normal control non-tumorigenic epithelial cell line-MCF10 were chosen and grown in 96-well plates and treated with WFs (10% WF/WF-RT in DMEM). To assess the effects of these WFs, we employed a 3-(4,5-dimethylthiazol-2-yl)-2,5-diphenyl-2H-tetrazolium bromide (MTT) assay, which indirectly measures cell growth. As shown in Fig. 2, three cancer cell lines incubated with WF, WF-RT, and DMEM (CTR) showed different viability after 24 h, 48 h, and 72 h of treatment. CTR cell lines showed 100% viability in all three incubation times, and WF groups were compared with CTR every time. MCF-7 represented the same result in both WFs-treated groups; however, after 48 h, the viability in WFs-treated groups was significantly increased compared to the CTR group. SKBR3 had significantly decreased viability just after 72 h incubation in WF-RT than the WF group. MDA-MB-231 showed a significant decrease in viability after 48 h incubation in WF-RT than in the WF group. According to Fig. 2, in cancer cells, abrogated proliferation was more in cells treated with WF-RT than WF after 48 h of incubation. The cell viability percentage in MCF-7 was more than MDA-MB-231 and SKBR3. In other words, the MDA-MB-231 and SKBR3 cell lines benefit more from the likelihood of anticancer effects of WF-RT. Toxicity responses of the normal-like cell line were different from breast cancer cell lines in treatment with WFs (WF and WF-RT). The cell viability of MCF10 increased in a time-dependent manner when treated with WF and WF-RT. There was no significant different response between DMEM, WF, and WF-RT at the same time, just at 72 h WF-RT significantly decreased the viability compared to WF-treated cells. Figure 2 also depicts the morphological changes of MCF-7 treated with WF and WF-RT compared with CTR, which shows higher cell confluency in CTR and WF compared to WF-RT. Our results revealed that in 2D, the cytotoxicity of WF-RT compared to WF depends on cell types and time of incubation, and the MDA-MB-231 cell line might benefit more from the likelihood of anticancer effects of WF-RT.

### 3D-tumor-derived spheroid after WF stimulation

**Figure 3:**
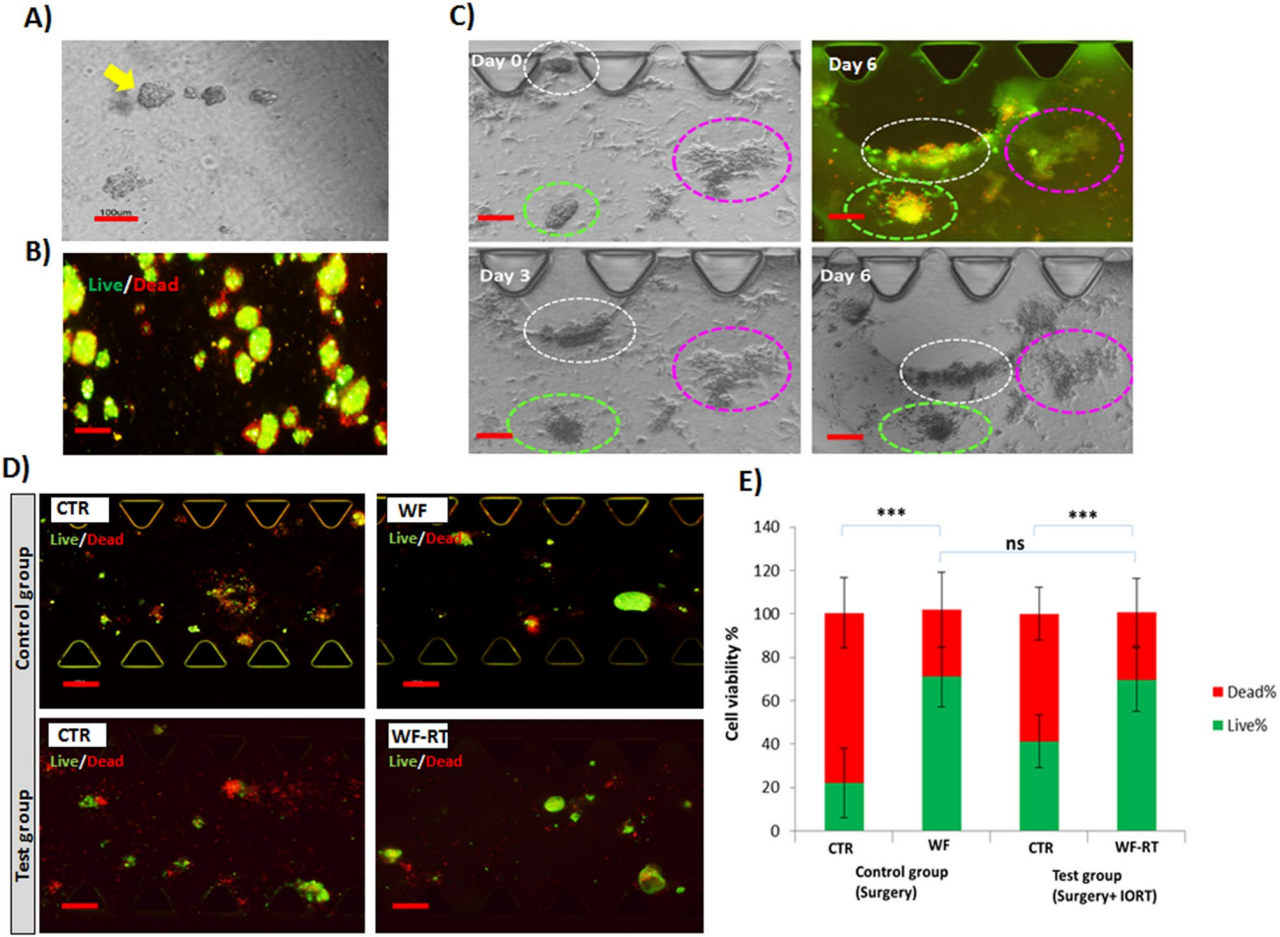
Optical and fluorescent images of spheroids in microfluidic devices on days 0, 1, 3, and 6 of culture. (**A**) Tumor spheroids are observed with a convert microscope before mixing with collagen at day 0. Scale bar: 100 µm. Original magnification: ×40. (**B**) Tumor spheroids stained with AO/PI before injection into the microfluidic devices to find spheroid viability on day 0. Scale bar: 100 µm. Original magnification: ×40. (**C**) The tumor-derived spheroids were treated with WF-RT on days 0, 3, and 6 with inverted phase-contrast microscopy and fluorescent microscopy. Different behaviors of three types of spheroids treated with WF-RT were traced for 6 days and shown with the circular dotted line. White dotted lines show motility of the spheroids during 6 days, while green ones represent the in-situ proliferation of cells within the spheroids and pink ones represent the spheroid without proliferation and motility. Scale bars: 100 µm. Original magnification: ×40. (**D**) live/dead staining of the spheroids under RPMI treatment comparing wound fluid treatment in a control sample (spheroids from the non-IORT treated patient) and a test sample (spheroids from IORT treated patient). Scale bars: 100 µm. Original magnification: ×40. Green: AO/live cells; Red: PI/dead cells. (**E**) The graph presents the comparison of %live and %dead cells in control samples with test samples and between RPMI and WF treated cells in each group. Control group: patients who only went under surgery, test group: patients who received IORT during the surgery. *CTR* control (RPMI + 10%FBS), *WF* wound fluid, *WF-RT* IORT-treated wound fluid. ns: non-significant. ***P < 0.001.

Here, we used AO/PI to stain and differentiate live from dead cells. The size of the tumor-derived spheroids was selected based on the size of the microfluidic devices' central channels^22^ and was verified by invert microscopy for all specimens (Fig. 3A). Spheroid viability was evaluated on day 0. Live cells (Acridine orange permeable) emitted a green fluorescent signal, while dead cells (Propidium iodide permeable) emitted a red fluorescent signal. Over 90% of the spheroids from each specimen were alive (Fig. 3B). Figure 3C depicts the part of 3D culturing of the patient-derived spheroids from WF-RT groups incubated for 6 days. The images show three different clones of the spheroid related to the rate of migration and proliferation. This figure clearly shows the changes in spheroid growth and motility in the collagen matrix and their viability after 6 days of incubation. Comparison between WF, WF-RT, and CTR is represented in Supplementary Fig. S1A,B. To evaluate the effect of IORT on wound fluid and tumor cell killing, tumor-derived spheroids containing RPMI treated cells and WF-treated cells in both postoperative WF and WF-RT groups were assessed by fluorescent microscopy on day 6 (Fig. 3D). As depicted in Fig. 3E, the analysis of live/dead staining on day 6 showed that the percentage of live cells in WF and WF-RT-treated spheroids were significantly more than the dead cells, whereas in the CTR-treated spheroids, the frequency of live cells dropped, and the percent of dead cells was increased. There was a significant increase in cell viability in wound fluid-treated spheroids in WF and WF-RT groups than RPMI-treated spheroids (P = 1.08E − 11 in the WF group and P = 1.90E − 07 in the WF-RT group). Furthermore, a comparison between the frequency of live cells in WF and WF-RT-treated spheroids revealed no significant difference between the two groups (P = 0.38).

### Clonal survival density was abrogated with WF-RT

**Figure 4:**
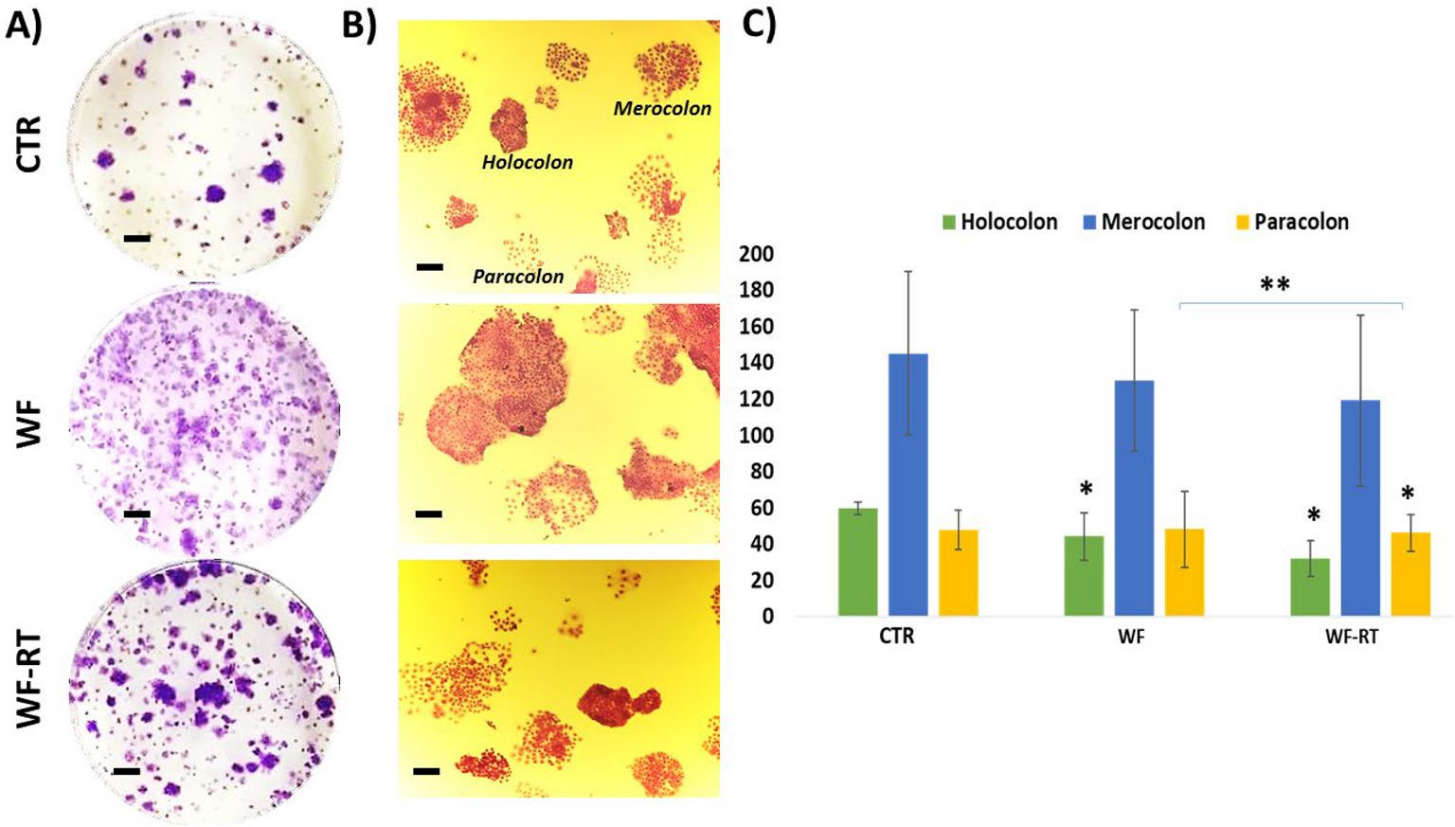
Clonogenic survival assays following treatment with WFs. MCF7 cells were treated with WFs from different groups. After 48 h, the compounds were removed, and cells were seeded at a density of 1000 cells for MCF7 on 35 mm plates. After 7 days of incubation, the cells were stained with crystal violet, and the stained plates were scanned. (**A**) Represents the images of colony density in a 6-well plate. Original magnification: ×40. Scale bars: 50 µm. (**B**) Images of colony shape. Original magnification: ×40. Scale bars: 100 µm. (**C**) Quantitative analysis based on colony shapes. Significant value from comparing WF and WF-RT groups with CTR groups has shown with *P < 0.05 and **P < 0.01.

In addition to the MTT assay, clonal survival assays were carried out to assess the survival and proliferative capacity of MCF-7 cells following treatment with WF-RT. MCF-7 cells were treated with WF and WF-RT and compared with CTR groups (DMEM-treated) for colony density and shape (Fig. 4A,B, respectively). WFs induced colony formation in a way that a higher level of colonies formed in WF than WF-RT groups. Figure 4C presents the number of holoclones, meroclone, and paraclone. The number of holoclones decreased significantly in WF and WF-RT-treated cells compared with the CTR. The number of paraclones also significantly decreased in WF-RT than in control and WF-treated cells.

### Inhibition cell signaling pathway-related cancer progression and invasion in the tumor margin

The upregulated and downregulated data obtained from the patient's tumor margin before and 24 h after IORT were represented in Supplementary Tables S2 and S3, respectively. In addition, KEGG pathway analysis based on upregulated and downregulated is also represented in Supplementary Tables S4 and S5 and, briefly represented in Tables 1 and 2, respectively^27^. The critical pathways suppressed by IORT were the PI3K-Akt signaling pathway, Rap1 signaling pathway, Focal adhesion, ECM-receptor interaction, Central carbon metabolism in cancer, and Glycolysis/Gluconeogenesis. In contrast, several pathways related to Carbon metabolism, Citrate cycle (TCA cycle), Fatty acid degradation, Valine, leucine, and isoleucine degradation, amino acid metabolisms such as Tryptophan, Beta-Alanine, Histidine, Arginine, and Proline were activated by IORT in tumor margin.

**Table 1:** KEGG pathway of downregulated proteins in tumor margin tissue treated with irradiated WF by DAVID enrichment database.

Term	Count	%	P-value	Genes
hsa04510:Focal adhesion	12	8.759124	3.24E − 05	P15498, P04004, P16234, P50552, P08514, P60709, P12814, P04275, P07996, P02751, Q9Y490, P21333
hsa04015:Rap1 signaling pathway	8	5.839416	0.013054	P16234, P50552, P08514, P05107, P60709, P11215, P07996, Q9Y490
hsa04512:ECM-receptor interaction	5	3.649635	0.020576	P04004, P08514, P04275, P07996, P02751
hsa05100:Bacterial invasion of epithelial cells	5	3.649635	0.0143	O15144, P60709, Q00610, O15511, P02751
hsa05230:Central carbon metabolism in cancer	4	2.919708	0.041693	P16234, P14618, P52790, P11413

**Table 2:** KEGG pathway of upregulated proteins in tumor margin tissue treated with irradiated WF by DAVID enrichment database.

Term	Count	%	P-value	Genes
hsa01200:Carbon metabolism	11	14.10256	8.49E − 09	Q6NVY1, P08559, P36957, O75874, P40925, Q02252, P11766, P05166, P50213, Q96I99, Q9P2R7
hsa00020:Citrate cycle (TCA cycle)	7	8.974359	7.17E − 08	P08559, P36957, O75874, P40925, P50213, Q96I99, Q9P2R7
hsa00071:Fatty acid degradation	8	10.25641	2.05E − 08	P42765, Q16836, P49189, P00325, P11766, P55084, P49419, P49748
hsa00280:Valine, leucine and isoleucine degradation	8	10.25641	4.64E − 08	P42765, Q16836, Q6NVY1, P49189, Q02252, P55084, P49419, P05166
hsa00380:Tryptophan metabolism	3	3.846154	0.036413	Q16836, P49189, P49419
hsa00410:beta-Alanine metabolism	5	6.410256	7.86E − 05	Q6NVY1, Q96KP4, P49189, Q02252, P49419
hsa00330:Arginine and proline metabolism	4	5.128205	0.006218	Q96KP4, P49189, P12277, P49419
hsa00340:Histidine metabolism	3	3.846154	0.011754	Q96KP4, P49189, P49419

### Cell cycle arrest and senescence in a 2D culture of cell lines after WF stimulation

**Figure 5:**
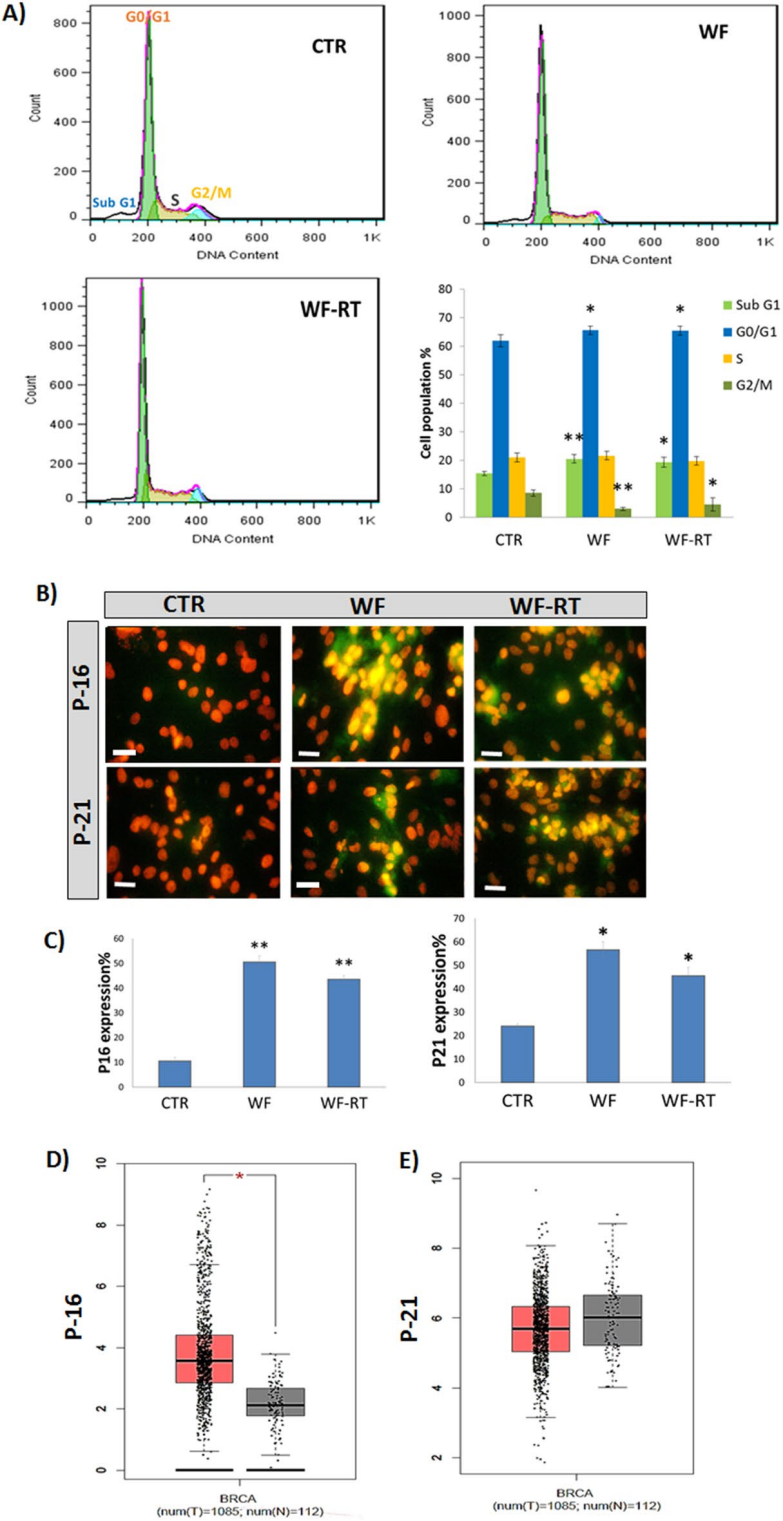
Effect of WFs on the cell cycle distribution of MDA-MD-231 cells. (**A**) Flow cytometry analysis for treated and untreated MDA-MB-231 cells. (**B**) Quantitative analysis of cell cycle arrest at the G0/G1 phase. Data represent mean ± SD of triplicate, *P < 0.05, and **P < 0.01. Scale bars: ×100. Original magnification: 10 µm. (**C**) The graphs represent the expression levels of P16 and P21 in MDA-MB-231 cells. The data represents the means ± standard deviations (SDs) of 3 independent tests. Green = P16 and P21, Red = PI. CTR: control (DMEM + 10%FBS), WF: wound fluid, WF-RT: IORT-treated wound fluid. (**D**, **E**) The expression levels of P16 and P21 in breast cancer and normal tissues were analyzed using GEPIA. In the box plots, the thick lines in the middle represent the median, and the upper and lower limits of the box represent the third and first quartiles, respectively. The top and bottom of the error bars represent the maximum and minimum data values, respectively; outliers were considered > 1.5 quartile spacing and excluded. *P < 0.05. *T* tumor, *N* normal, *num* number.

PI staining and flow cytometry analysis was performed to investigate the cell cycle phase distribution in MDA-MB-231 cells after 48 h of treatment with WF/WF-RT (Fig. 5A). According to the results based on flow cytometry analysis, the treatment of WF and WF-RT caused a significant increase in the sub-G1 cell population (apoptotic cells) compared with control. Furthermore, a significant increase in the number of G0/G1 of WF and WF-RT-treated cells were detected compared to control DMEM-treated cells (CTR). The percentage of cells in the G2/M phase was decreased in WF and WF-RT-treated cells, and the results were statistically significant. The results indicate that the WF and WF-RT cells arrested the cell cycle in the G0/G1 phase and induced apoptosis after treatment for 48 h. Cells treated with WF and WF-RT showed senescence after 3 days than that of CTR group, which showed cell proliferation. Hence we evaluated the expression of two proteins related to the senescence, P16, and P21, in MDA-MB-23 treated with WF and WF-RT and compared them to CTR. Figure 5B,C show the immunocytochemistry images and the graphs related to differential expression of P16 and P21. The results showed a significantly increased expression of proteins, P16 and P21, in WF and WF-RT-treated cells compared with the CTR group. There was no significant difference in the expression level between WF-treated and WF-RT-treated cells. We did not see any senescence condition in the 3D spheroid after 6 days of incubation. Figure 5D,E show the P16 and P21 mRNA expression with GEPIA analysis that indicated expression of P16 was significantly higher in breast cancer than in normal tissues, whereas P21 was observed lower breast cancer than in normal tissues but was not statistically significant differences (Fig. 5E).

### Increased apoptotic process in cell lines and spheroids after WF stimulation

**Figure 6:**
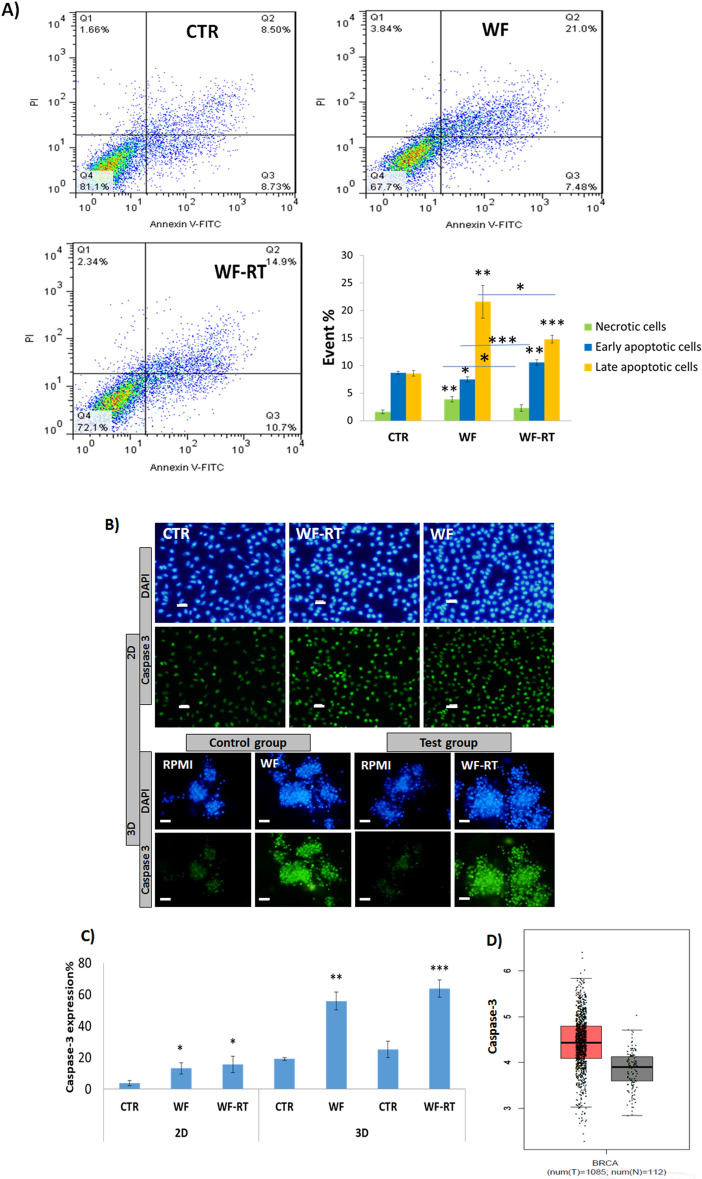
Apoptotic assays in BC cell lines and human-derived tumor spheroids. (**A**) Annexin V-FITC and PI staining to evaluate apoptosis in MDA-MB-231 cells following WFs treatment. MDA-MB-231 cells were treated with WF-RT (10% in DMEM, for 48 h), incubated with Annexin V-FITC and PI, and analyzed using flow cytometry. In each panel, the lower left quadrant shows cells, which are negative for both PI and Annexin V-FITC, upper left quadrant shows only PI-positive cells, which are necrotic. The lower right quadrant shows Annexin-positive cells (early apoptotic), and the upper right quadrant shows Annexin and PI-positive cells (late apoptosis cells). The percentage of necrotic, early, and late apoptotic cells are represented in the graph. (**B**) In 2D culture, MDA-MB-231 cells were cultured and treated with WF and WF-RT for 48 h. The expression level of Caspase 3 significantly increased in WF and WF-RT treated cells compared with DMEM-treated cells (CTR). Original magnification: ×40. Scale bars: 10 µm. In 3D culture, human-derived tumor spheroids were cultured and treated with RPMI, WF, and WF-RT for 6 days. The expression level of Caspase 3 in WF and WF-RT-treated cells was significantly increased compared with RPMI-treated cells (CTR). Original magnification: ×100. Scale bars: 50 µm. This increase was more significant in the WF-RT group than others. (**C**) The graph related to the expression level of Caspase 3 shows no significant difference between the IORT and non-IORT groups. (**D**) Expression of Caspase-3 in breast cancer and normal tissues analyzed using GEPIA. In the box plot, the thick line in the middle represents the median, and the upper and lower limits of the box represent the third and first quartile, respectively. The top and bottom of the error bars represent the maximum and minimum data values, respectively; outliers were considered > 1.5 quartile spacing and excluded. *P < 0.05, **P < 0.01 and ***P < 0.001. Control group: patients who only went under surgery, test group: patients who received IORT during the surgery. CTR (control): DMEM in 2D/RPMI in 3D, WF: wound fluid, WF-RT: IORT-treated wound fluid, T: tumor; N: normal; num: number.

Since the cell viability test showed different cytotoxicity in different cell lines, we were interested in examining apoptosis induction by Annexin V-FITC/PI staining. MDA-MB-231cells were treated with WFs (10% in DMEM + FBS + pen/strep for 48 h) and stained using Annexin V-FITC/PI. Regarding Fig. 6A, the flow cytometry analysis of MDA-MB-231cells pointed out that through the treatment with WF and WF-RT-treated, the cell population shifted from viable to apoptotic. After treatment with WFs, there is also a significant difference between early and late apoptosis and necrosis in the WF-RT-treated group with the WF-treated group. These results describe the ability of WF-RT to induce apoptosis, expressly in early-stage apoptosis in MDA-MB-231 cells. Furthermore, to understand whether irradiated wound fluid can impact the expression level of the apoptosis-related enzyme caspase 3 in MDA-MB-231 cells and tumor-derived spheroid in the microfluidic system, we compared the immunocytochemistry assay results between the WF and WF-RT groups and compared them with CTR. Figure 6B represented the caspase-3 expression in 2D and 3D culturing of breast cancer. In 2D and 3D, the expression level of caspase 3 was significantly increased in both WF-treated and WF-RT-treated cells and spheroids compared with CTR. WF-RT-treated cells contain a higher level of caspase-3 expression than WF-treated cells but showed no significant difference (Fig. 6C). To find the caspase-3 mRNA expression in cancer tissue compared to normal breast tissue, we used GEPIA analysis that retrieved data from TCGA. The result indicated that caspase-3 expression was higher in breast cancer than in normal tissues (Fig. 6D) but was not statistically significant.

### Evaluation of migration and invasion in cell lines and spheroids: 2D vs. 3D

**Figure 7:**
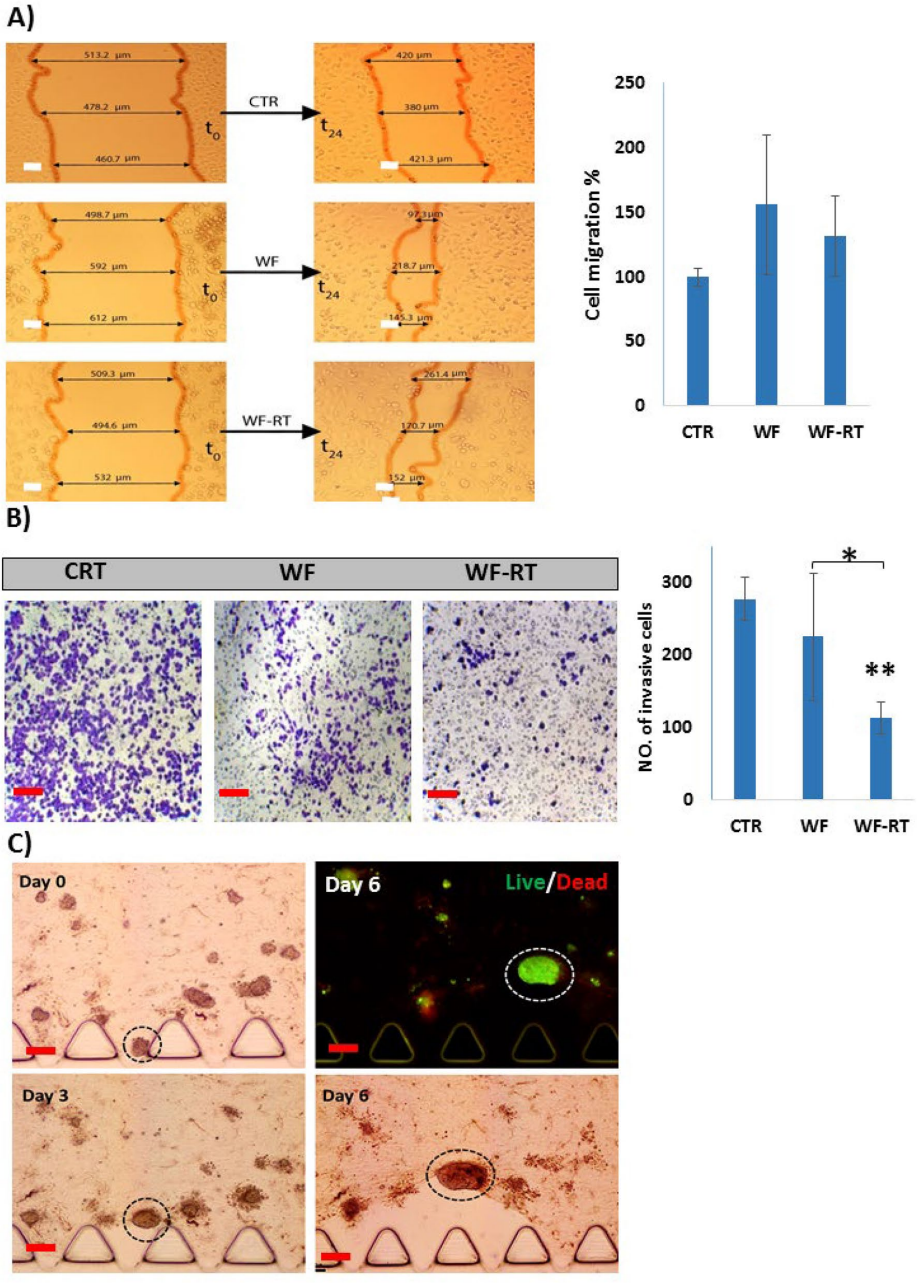
Effects of WFs on migration and invasion BC cell line (MDA-MB 231) and in human-derived tumor spheroids. (**A**) Images and graphs related to scratch assay (wound healing compared to negative control in 0 and 24 h). The graph presents the percentage of migrated cells. (**B**) Images of the cells on the upper chamber in transwell assay and graph present the percentage of cell migration. *P < 0.05, and **P < 0.01. (**C**) Migration of BC tumor spheroids in microfluidic devices. Circular dotted lines show one of the spheroids that migrate during 6 days of incubation with WF. Scale bars: 100 µm. Original magnifications: ×40.

To assess the effects of WF-RT on migration and invasion of MDA-MB-231cells, wound-healing assay and transwell assay were employed, respectively. As shown in Fig. 7A, the wound-healing assay indicated that WF-RT and WF induce migration of MDA-MB-231 cells compared with CTR groups, but the difference is not significant. Besides, WF-RT abrogated the migration compared to WF. The transwell invasion assay is also designed to assess the ability of MDA-MB-231 cells to invade through the Matrigel. As shown in Fig. 7B, WFs almost inhibited cell invasion, and this inhibitory effect was higher in cells treated with WF-RT than WF; WF-RT could significantly inhibit the invasion compared with CTR and WF. Migration and invasion in the 3D culture of spheroid-derived patients with different tumor microenvironments depended on several factors seen in some tumor spheroids that variably respond in the presence of WF/WF-RT. As observed in Fig. 7C, one of the spheroids migrated after 6 days of WF incubation, while most have in situ proliferation without migration. Figure 3C also showed a single live spheroid treated with WF-RT and was migrated after 6 days of culture. Supplementary Fig. S2a,b represent the migration of spheroids treated with RPMI(CTR) in both samples of patients who received only surgery (a) and surgery plus IORT (b). However, after 6 days of culture, more migration was observed in the 3D spheroid system treated with WF/WF-RT. Interestingly, the spheroids with higher migration were accompanied by a wrap of collagen around them that could move with the spheroids.

**Figure 8:**
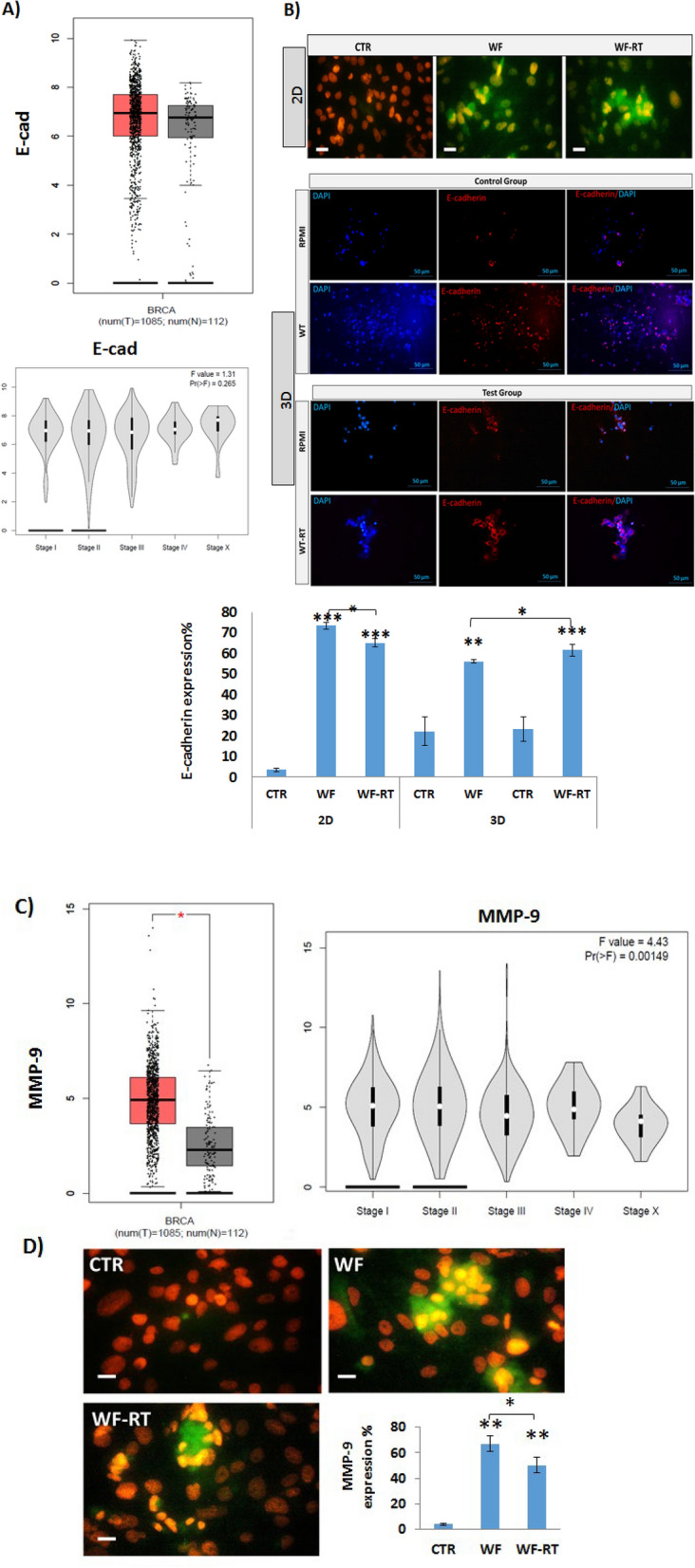
(**A**) Association of mRNA expression of E-cad and tumor stages in patients with breast cancer analyzed using GEPIA. In the violin plots, the white dots represent the median; the black bars represent the 95% confidence intervals; the black lines represent the interquartile range, and the gray shapes' width represents the distribution density. F-value, the statistical value of F test; Pr (> F), P-value. (**B**) Immunocytochemistry analysis of MDA-MB-231 cells following WFs treatment, including the expression levels of E-cadherin in 2D and 3D systems and the average percent of E-cadherin represented in WF and WF-RT and CTR groups. Green: E-cadherin and MMP9, Red: PI. Scale bars: 10 µm in 2D, 50 µm in 3D, magnification: ×100. (**C**) The expression of MMP-9 in breast cancer and normal tissues were analyzed using GEPIA. In the box plots, the thick line in the middle represents the median, and the upper and lower limits of the box represent the third and first quartile, respectively. The top and bottom of the error bars represent the maximum and minimum values of data, respectively; outliers were considered to be > 1.5 quartile spacing and were excluded. *P < 0.05. T, tumor; N, normal; num, number. (**D**) In 2D culture, the expression level of MMP-9 significantly increased in both WF and WF-RT treated cells compared to CTR. *P < 0.05, **P < 0.01 and ***P < 0.001. Scale bars: 10 µm. Original magnification: 40X. Control group: patients who only went under surgery, test group: patients who received IORT during the surgery.

E-cadherin expression was analyzed to assess the molecules inhibiting the cells' invasion properties. E-cadherin expression's violin and box plot were acquired from the GEPIA database. This database compares the gene expression of normal and breast cancer tissues retrieved from the TCGA database. The results revealed the higher expression of E-cadherin, although there was not a significant difference in cancer tissue compared to normal tissue. The violin plot confirmed the higher expression of the gene in the early stage than the late stage (Fig. 8A). To understand whether irradiated wound fluid can impact the expression level of the EMT-related E-cadherin, we compared the immunocytochemistry assay results between WF and WF-RT groups in 2D and 3D. Further, results obtained from WF and WF-RT groups for each sample were compared to RPMI-treated spheroids (CTR) (Fig. 8B). Our data revealed that the molecule inhibiting the cells' invasion property, E-cadherin, was increased in both WF-treated groups. When comparing the 2D MDA-MB-231 culture system with the 3D tumor-derived spheroid, the CTR groups in 2D had a much lower level of expression E-cadherin than 3D. The expression level of E-cadherin was significantly increased in WF-treated MDA-MB-231 and spheroids compared with CTR groups. This difference is more significant in the WF-RT group (P = 9.45E − 05) than in the WF group (P = 0.006). However, when considering E-cadherin, results obtained from WF and WF-RT spheroids indicated that E-cadherin expression level was significantly (P = 0.014) increased in WF-RT-treated compared to WF-treated spheroids; whereas, no difference was noted when analyzing CTR spheroids (P = 0.41) in both groups. Our previous proteomic analysis by ITRAQ technique on patient tumor margin incubated with RT-WF for 24 (compared to before incubation) also demonstrated a higher significant level of E-cad expression (Mean-Ratio:1.22, Q-value < 0.05). However, there was no significant differential E-cad expression between tumor tissues and their margins. Between the cadherin superfamily, CAD13 was detected overexpressed in the tumor (Mean-Ratio:2.04, Q-value < 0.05) with gene ontology related to the cancer progression process such as negative regulation of cell adhesion, positive regulation of cell migration, and positive regulation of endothelial cell proliferation. MMP-9 mRNA expression is associated with breast tumor tissue compared to normal tissue obtained from GEPIA analysis indicated that expression of MMP-9 was significantly higher in breast cancer than in normal tissues. The Violin plot also represented the higher level of expression in the late stages (Fig. 8C). In 2D cell culture, we assessed the MMP-9 in MDA-MB-231cells and found that expression was significantly increased in WF and WF-RT-treated groups compared with DMEM-treated cells (CTR). In addition, MMP-9 expression in WF-RT was significantly decreased compared to WF-treated cells (Fig. 8D). In our previous study and analysis of ITRAQ data, we found that level of MMP-9 expression was significantly lower in tumor margins incubated with RT-WF for 24 h compared to before incubation (Mean-Ratio: 0.19, Q-value < 0.01)^14^.

### Schematic Illustration of the direct and bystander effect of IORT

**Figure 9:**
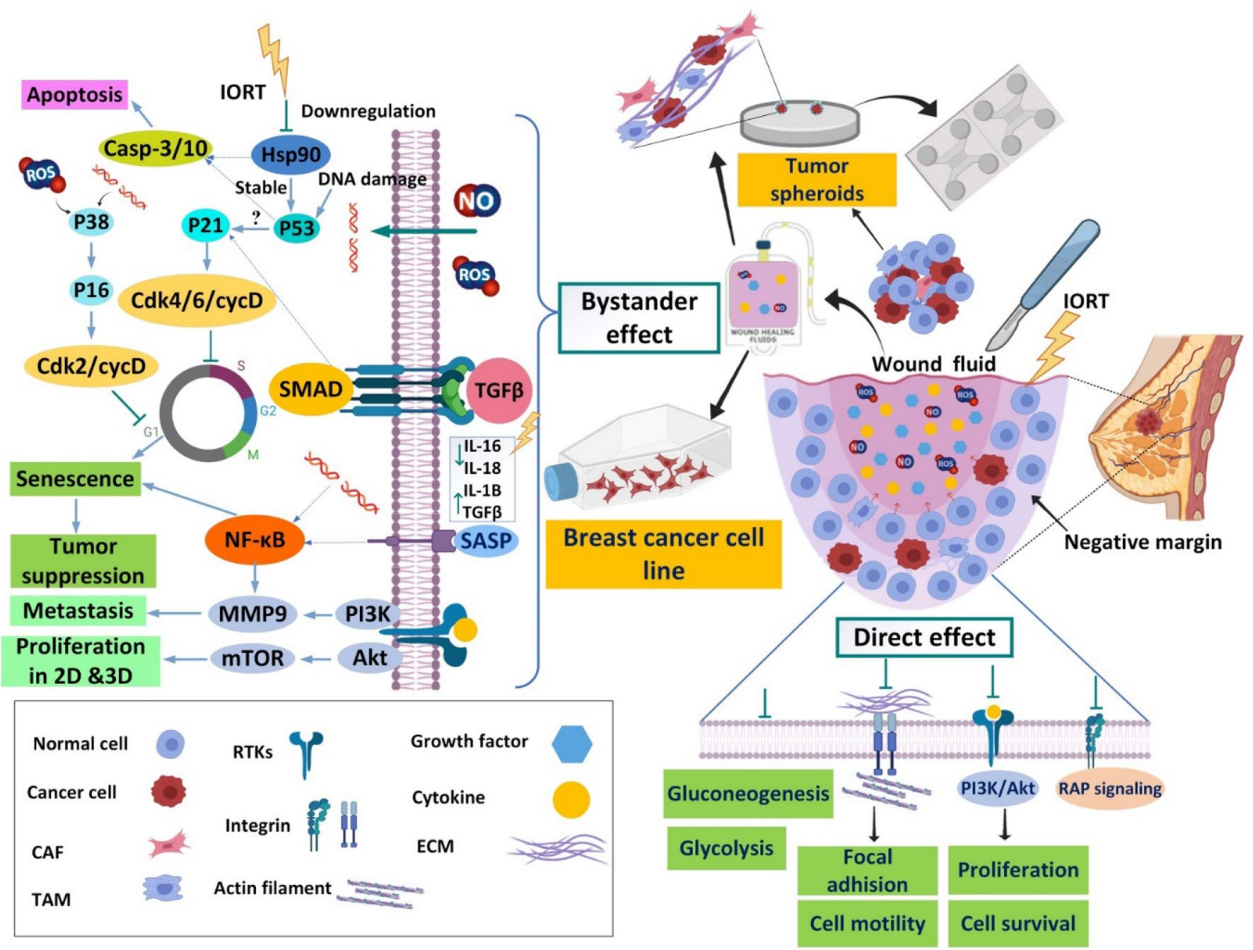
Proposed schematic showing direct and bystander effects of WF of IORT. After surgery, tumor spheroids were established to study the tumor behavior in 3D culturing in tumor on-chip. Tumor spheroids contain many cell types, cancer cells, cancer-associated fibroblasts (CAFs), and Tumor-associated macrophages (TAMs). Negative margin exposure to IORT shows the direct effect of radiation that inhibits several signaling pathways that its effect could target the residual cancer cells in the negative margin. Wound fluid (WF) produced in the surgical cavity contains several cytokines, growth factors, and free radicals after IORT, which have a bystander effect. After 24 h of IORT, WF was collected and studied on 3D spheroid and 2D cell culturing that show several biological processes, including arrested cell cycle through free radicals and DNA fragmentation, downregulation of Hsp-90 that stabilized TP-53 in cancer cells, upregulation P21 and P16 that promote cell cycle arrest and finally senescence. Senescence-associated secretory phenotype (SASP) factors also activated pathways to inhibit the cell cycle and activated several biological processes such as apoptosis and senescence. Growth factors were increased to promote the wound healing process in the surgical cavity after IORT and increase proliferation, motility, and migration of some spheroids and cell lines. The figure was created using Biorender (https://biorender.com).

After surgery, tumor spheroids were established to study the tumor behavior in 3D culturing in tumor on-chip. Tumor spheroids contain many cell types, cancer cells, cancer-associated fibroblasts (CAFs), and Tumor-associated macrophages (TAMs). Negative margin exposure to IORT shows the direct effect of radiation that inhibits several signaling pathways, the PI3K-Akt signaling pathway, Rap1 signaling pathway, Focal adhesion, ECM-receptor interaction, Central carbon metabolism in cancer, and Glycolysis/Gluconeogenesis. In the direct effect, radiation targets the residual cancer cells in a negative margin. Wound fluid (WF) produced in the surgical cavity contains several cytokines, growth factors, and free radicals after IORT, which have a bystander effect. After 24 h of IORT, WF was collected and studied on the 3D spheroid, and 2D cell culturing, showing numerous biological processes, including arrested cell cycle through ROS and NO that cause several damages, DNA fragmentation, and activated P38 to increase P16. Downregulation of Hsp-90 that stabilized TP-53 in cancer cells could activate the apoptosis process. Upregulation P21 and P16 promote cell cycle arrest and finally cause senescence. WF secret senescence-associated secretory phenotype (SASP) factors such as TGF-b also activated pathways to inhibit the cell cycle and activated several biological processes such as apoptosis and senescence. IL-1 also increased in RT-WF that activated inflammatory cascade helping promote senescence, which leading to tumor suppression. However, increased growth factors produced to accelerate the wound healing process in the surgical cavity after IORT cause increased proliferation, motility, and migration of some spheroids and cell lines (Fig. 9).

## Discussion

Mastectomy provokes an acute wound that the residual tumor cells may persevere within negative excision margins by automatically imperiling the WF, raising the risk of recurrence. At the same time, there is substantial evidence supporting the efficacy of IORT^23,28^. Likewise, trials have shown that adjuvant radiotherapy controls the loco-regional recurrence^7,29^ and has an abscopal effect by reducing the risk of distant recurrence^24,25,30,31^. Our previous proteomic and transcriptomic study on the samples of tumor bed before and after IORT illustrated many gene expression modifications that enriched in pathways related to cell growth, survival, program cell death, and cell cycle arrest^14^. In the present study, we evaluated the radiobiological effects of wound fluid on tumor spheroids in the microfluidic system and compared the results with 2D monolayer assays in BC cell lines. Recently, Kulcenty et al. reported that molecular subtypes of breast cancer have a different response to IORT^17^. In 3D systems, WF stimulates cancer cell proliferation, and there was no significant difference between the treatment of the cells with WF and WF-RT. The first and only report associated with the WF effect from TARGIT-treated and untreated patients investigated the 3D culture of MCF-7 breast cancer cell line implied that TARGIT treatment impairs the cancer cell growth in 3D matrices^15^. This research employed only one breast cancer cell type to form spheroid to further assess the effect of WF from TARGIT-treated in 3D cell culture while we used the patient-derived tumor spheroids in combination with different cells located in tumor bulk. Here, with the different cell types in tumor spheroids, the cells that grow in response to both WF and WF-RT might be related to the supporting function of stroma cells. Although colony formation assay showed the simulative proliferation was more remarkable in WF/WF-RT treatment, a decreased number of holoclones (Fig. 4) is regarded as enrichment for cancer stem cells and self-renewal property^32^. This data agrees with previous studies showing a significant decrease in the population of breast cancer stem cells in the WF-RT treated group^33^. In addition, WF-RT induced a significant number of paraclones in MCF-7 compared to WF and CTR. Paraclones with a differentiated morphology have a minimal proliferative potential and little self-renewal capacity^32^. So WF-RT could diminish the cancer stem cell capacity in treated cancer cells. These findings confirm the hypothesis that surgical wounds can remodel the tumor microenvironment, provoke tumor growth and develop local recurrence by activating the proliferation and motility of remaining cancer cells, whereas some of these results were abrogated by WF-RT^1,15^.

Growth factor-mediated signaling such as the PI3K/AKT/mTOR signaling pathway is responsible for cancer proliferation and cell survival^34^. We found downregulated proteins belong to the PI3K-Akt signaling pathway that shows disruption of proliferation after radiation (Table 1). Furthermore, inhibition of this pathway was detected in radiation's direct and indirect effects^14,35^. Crosstalk between growth factor-mediated signaling(PI3K/AKT/mTOR) and cell adhesion to the ECM contributes to the remarkable critical biological processes, including cell proliferation, cell differentiation, cell motility, regulation of gene expression, and cell survival^36,37^. By direct radiation of the margin of tumor after 24 h of BCS plus IORT, we found downregulated proteins in KEGG pathway analysis enriched in process related to focal adhesion, ECM-receptor interaction, and Rap1 signaling pathway. Rap1 is a small GTPase that controls diverse processes, such as cell adhesion, cell–cell junction formation, and cell polarity^38^. Beforehand proclaimed, ECM composition adjusts the radiation sensitivity of cancer cells ^39^; it also modifies the ECM of normal tissue by producing fibrosis^40,41^. The bystander effects interfere through gap junction connection or bystander signals from neighboring irradiated cells^42^. Principally cytokines and chemokines are responsible for this effect^43,44^. Consequently, in addition to its direct action on remaining tumor cells in the margin, the bystander effects of irradiation may also be recognized in non-irradiated cells located close to the tumor site, including a significant alteration of the tumor microenvironment. Furthermore, central carbon metabolism in cancer is also downregulated after IORT in tumor margin after 24 h.

Cancer cells modify their metabolism to maintain unlimited cellular proliferation and respond to the enhanced energetic and biosynthetic demands^45^. For example, they have an increased demand for glucose, so enhancing the glycolysis process that was first discovered by Warburg^46^. After radiation, process-related Glycolysis/Gluconeogenesis was inhibited (Table 1) and subsequently activated several carbon metabolisms such as citrate cycle, fatty acid, and amino acid degradation. These alterations in metabolism could provide the environment to inhibit the proliferation of residual tumor cells following IORT and attract immune cell infiltration^47^. Modified margin and tumor microenvironment mediated by IORT secrete different regulatory molecules that suppress tumor cell growth in situ*,* and this outcome was associated with the beneficial effect of IORT in patient survival^14^. However, at the same time, this secretome could induce tumor growth in vitro and ex vivo.

Nowadays, growing evidence indicates that cell death induction is a complex mechanism for radiotherapy therapeutic effects. Notably, in the last decade, it is becoming more apparent that inhibiting tumor cells proliferative capacity after irradiation can occur through different ways such as autophagy, necrosis, apoptosis, mitotic catastrophe, and senescence^29,48^. Here, we evaluated factors related to cell cycle arrest. Although the difference in the percentage of cells treated with WFs and CTR group was not large, we found that WFs significantly increased sub-G1 indicated the cells underwent apoptosis and were arrested in G0/G1 phases. Regulation of G1 phase progression is mediated by growth factors^49^, and analysis of human wound fluid has revealed some growth factors^50^, many of which are known to be directly mitogenic for tumor cells^51^. The effects of typical indirect radiation occur through the formation of free radicals and bystander responses, which happen in cells not directly irradiated but exchange signals with the irradiated. Secretory signals produced by the juxtaposing irradiated cells mediate the bystander effects. They can be related to ROS, NO, and calcium fluxes. Some cytokines may also act as mediators of bystander response, such as TNF-alpha, IL-8, and TGF-beta. This senescence-associated secretory phenotype (SASP) could induce paracrine senescence through DNA damages and arrest cells in G0/G1 phase^52^. DNA damage detection may cause cells to leave the cell cycle or undergo apoptosis, consequently not to reproduce irreparably damaged chromosomes^53^. A previous study showed that cell-cycle checkpoint and DNA repair processes are higher in RT-WF than in cells stimulated with WF alone. In addition, cell cycle regulatory genes such as CDK2 significantly decreased in WF-RT compared to WF^54^. We also found a significant increase in CDK inhibitory gene expression, P21, which induced G1-phase arrest in MDA-MB-231 cells. However, we found no significant difference of G0/G1-arrest between WF treated cells, and X-ray-IORT WF treated cells; while in our previous study, we have observed a significant increase in the G0/G1 phase of MDA-MB-231 treated with WF from patients who received electron IORT (21 Gy) (unpublished data). This finding indicated that WF from electron IORT could arrest cell cycle more significantly than X-ray IORT compared to surgery WF. In addition to the type of IORT source, previous studies have shown that IORT treatment affects WF composition in breast cancer patients depending on their molecular subtype^17^.

Bystander senescence is a potentially significant effect on IR exposure cells^55^. Senescence is caused by the permanent cessation of the cell cycle in the G1 stage. It is often associated with an increase in expression of P16INK4A (CDKN2A) and P21 (CDKN1A, WAF1/CIP1), known as cell cycle inhibitors^56^. We found that wound fluid (WF/WF-RT) increased P16 and P21 protein expression levels in MDA-MB-231 cells. P21 protein induced by senescence-associated secretory phenotype (SASP) through TGFb-SMAD signaling pathway, and it is a highly p53-transactivated gene^57^. It is a CDK inhibitor that binds to PCNA to prevent cells from entering the S phase. One primary role of P21 appears to be G1 phase arrest mediation. It also has essential roles in aging, regulation of apoptosis, and differentiation^58^. After radiation, the wound healing process triggers the secretion of several factors such as inflammatory cytokines, growth factors, and matrix metalloproteinases as part of SASP^59^. These bystander signals could induce cellular senescence in the tumor bed that finally lead to tumor suppression^60^. X-ray irradiation can induce double-stranded DNA breaks and lead to a cascade of p53-dependent signal transduction^61^. Elevated p53 levels may contribute to an arrest of the cell cycle in G1 or cell apoptosis. DNA damage can lead to a signal transduction pathway that can lead to an increase in p53. However, this happens by stabilizing an existing protein instead of increasing its transcription^62^. MDA-MB-231 is a human breast cancer cell line with high levels of a type of mutant p53^63^ containing HSP90, which might stabilize mutant p53 protein and lead to cell survival^64^. On the other hand, MDA-MB-231 irradiated cells represented the downregulation of HSB90 isoforms, causing increased apoptosis. So, WF-RT could induce apoptosis through pathways related to the P53 destabilization or independent P53 pathways. In addition to flow cytometry assays that showed both WF and WF-RT increased early and late apoptosis in MDA-MB-231 cells, we evaluated the expression levels of Caspase 3 that significantly increased in WF/WF-RT. In tumor spheroids, Caspase3 also increased in response to WFs, and we did not identify a significant difference between WF and WF-RT effects, although the amount of expression in WF-RT was higher than WF. Several studies have shown a correlation between the down-regulation of Caspase-3 and the development of breast cancer^65–68^. Results of the cancer atlas revealed that Caspase 3 has higher expression but is not significant in breast cancer than normal tissue. A meta-analysis study on more than 3000 breast cancer cases showed that overexpression of Caspase-3 is significantly associated with poor overall survival^69^. In the present study, we noted that the levels of Caspase-3 are increased in spheroids undergoing WFs treatment compared with spheroids exposed to medium only. However, there was no significant expression level of Caspase3 between irradiated and non-irradiated groups. Our results did not confirm the reported output from Kulcenty and colleagues. Although they did not report evaluated caspase3, they found that IORT-derived wound fluid activates extrinsic apoptosis pathways in the MCF-7 cell line through increasing Caspase 10^70^. A recent study on breast cancer cell lines also revealed that IORT-WF-treated cells show a higher level of breaks in double-strand DNA than cells treated with wound fluid plus conditioned media. The authors also observed that apoptosis increased in triple-negative cells affected by IORT-treated WF^16^.

EMT causes metastasis, and chemoresistant properties are connected to acquiring more stem-cell-like characteristics, causing increased migration and invasion capabilities^71^. Given that the mesenchymal cells are identified by improved motility, we first performed an in vitro migration and invasion assay (i.e., the scratch assay and transwell cell invasion) to functionally verify EMT transition. Afterward, we analyzed the expression of epithelial markers, E-cad, and MMP-9 in MDA-MB-231cells incubated with WF/WF-RT groups. We also tracked the migration of spheroids and analyzed E-cad in the 3D spheroid system. We observed a more mesenchymal phenotype in WFs-treated cells due to more migration in WF/WF-RT than CTR, but the difference was not significant. However, cells incubated with WF migrated much faster than RT-WF treated cells (Fig. 7). In 3D, some of the spheroids in WFs groups showed more migration than CTR groups. We also demonstrated that cells incubated with WFs abrogated invasion in 2D cell culturing and that the invasion effect was significantly suppressed in RT-WF treated cells than WF treated cells (Fig. 7). The study by Kulcenty K et al. observed induction of EMT process in 2D culturing of cells after incubation with WF/WF-RT that abrogate EMT by WF-RT was only statistically significant in the MDA-MB-468 cell line^72^. However, this finding is compatible with our data; remark that they used electron IORT, whereas we used photon energy for IORT delivery. Another study by Belletti et al. stated that the 3D culture of the MDA-MB-231 cell line invaded much faster the following incubation with WF from patients after BCS than WF-RT^15^. We observed different results of migration capability in tumor-derived spheroid with more migration in WF/WF-RT than CTR groups, and this stimulatory effect is not seen in all spheroids of one sample. In a microfluidic device observed, a small number of spheroids migrated. ECM composition inside tumors is heterogeneous, affecting cell behavior and cell fate, contributing enormously to tumor cell heterogeneity and EMT^41^. Previously Wei et al. stated that increasing stiffness of the surrounding ECM induces EMT in breast cancer cells by helping TWIST1 translocalization into the nucleus^26^. Tumor-associated fibroblasts (CAFs) are the primary source of the ECM in tumor-derived EMT^73,74^. The similarity of Belletti et al. and Kulcenty K et al. and our opposite 3D results might be related to this heterogeneity that emerges in spheroids. Culturing tumor-derived spheroid in microfluidic devices could provide the condition for in vivo modeling of tumors. The breast tumor-on-chip model used here recreates a real-like tumor environment for the remaining spheroids' growth and behavior in the tumor cavity after the BCS. Also, patient-derived tumor spheroids were composed of cancer cells, immune cells, tumor-associated fibroblasts, etc., that interact with each other to respond to wound fluid content. The tumor microenvironment is the indirect target of IORT that could change by bystander effect. Some in vitro studies pioneered by Belletti and colleagues suggested changes in the molecular composition of wound fluid following IORT inhibit EMT, that this effect is more pronounced in basal-like breast cancer cells. However, Kulcenty et al. believed that the source of energy during IORT treatment does not modify the biological characteristics of the WF. Therefore, we propose that the study of WF can be influenced by the type of in vitro tumor models because Belletti et al. also used WF from photon IORT to affect the 3D culturing of MCF7, T47D, and MDA-MB 231, and their results were different from our results that 3D tumor-derived spheroid was under treatment with WF from photon IORT.

We also assessed E-cadherin expression level to evaluate migration and invasion of the cells under treatment of WFs. We observed that in both 2D and 3D-microfluidic systems, WFs increased the expression level of E-cadherin. Interestingly, in the 3D model, we observed that IORT-wound fluid increases E-cadherin expression to a greater extent than non-IORT wound fluid, whereas; this finding was adverse in the 2D system. Our previous proteomic analysis by ITRAQ technique on patient tumor margin incubated with RT-WF for 24, proteome compared with before incubated tissue group, also demonstrated a higher significant E-cad expression level^14^. E-cadherin is a critical protein for epithelial cell–cell adhesion^75^ was strongly expressed on noncancerous epithelial cells, whereas various expression patterns were found in invasive and noninvasive breast carcinomas and correlations with clinicopathological features^76^. Analysis using the GEPIA bioinformatics tools retrieved E-cad expression from the TCGA database also emphasizes our finding because our patients were in stages 1 and 2 with an E-cad expression seen in CTRs. Since the patients belonged to invasive ductal carcinoma, most expressed E-cadherin in tumor tissue at high levels^77^. In contrast, we observed the lowest E-cad expression in 2D analysis and previously determined the negative expression of E-cadherin in MDA-MB-231 as a metastatic cancer cell line model^78^. Besides, in our previous proteomic analysis, we found that E-cad expression was not significantly different in tumor tissue than its margin. This data shows similar expression in tumor tissue and its margin and the result detected in the boxplot and violin plot obtained from the TCGA database. A comparison between 2D cell culturing and 3D spheroid implies the inherent difference between 2D cell line models and 3D spheroid composed of different cell types when investigating toxicology, migration, and invasion.

Matrix metalloproteinases (MMPs)-caused fragmentation of ECM components^79^ that progressed after radiotherapy preceding increased migration, angiogenesis, and metastasis^80^. In addition, the co-culture of squamous cell carcinoma cells with irradiated fibroblasts recognized increased invasiveness associated with the magnifying expression of MMP-9^81^. MMP-9 is significantly induced in breast cancer tissues and here also shows the increasing levels of expression in MDA-MB-231 cells when treated with WFs. However, there is a higher significant expression in WF/WF-RT than CTR; WF-RT significantly declined this effect compared to WF. NF-κB creates resistance to radiotherapy by producing MMP2/9^82^. The signaling pathway members were upregulated in the IORT of tumor bed^14^, so NF-kB can cause enhancing MMP-9 in cells affected by WF. A previous study also reported that IL-6 induces expression of metastasis modulators MMP-9 and MMP-2^83^, and WF composition contains a high level of IL-6 whereas decreased by TARGIT^15^. In addition, it was indicated that expression of MMP-9 is increased by mediated of EGF (epidermal growth factor) in ovarian cancer cells^84^. So, WFs contain several growth factors secreted in the wound healing process after surgery that could stimulate the expression of MMP-9. The safety of early drain wound fluid in breast cancer patients has been investigated in multiple studies. A randomized trial reported a significant improvement in the quality of life and clinical outcome in breast cancer patients undergoing early drain removal^85^. Studies on primary breast cancer cells indicate that wound fluid promotes cell chemoresistance^86^. Further, the expression level of pro-oncogenic cytokines and growth factors in surgical-induced wound fluid differ significantly between benign and malignant lesions^87^. Several reports have indicated that the accumulation of surgery-induced wound fluid in the surgical cavity after lumpectomy stimulates wound healing processes, which likely contribute to the increased risk of local recurrences in patients with breast cancer^88–90^. More investigations are required to decide if the RT-WF is a risk factor for cancer recurrences, as seen for WF. Finally, We concluded that most of the beneficial effects of IORT might be related to changes in regulatory elements due to IORT responsible for bystander and abscopal effects, which could not model in 2D cell culture and this kind of tumor-on-chip device. We could show the interaction between wound healing factors and patient-derived spheroids secreted either after surgery and IORT. These opposite results in 2D and 3D might be related to the heterogeneity that emerges in spheroids that microfluidic devices could fine-tune for in vivo modeling of tumors. This data need to be repeated in more different subtypes of breast cancer cell populations. To the best of our knowledge, there was no study on the impact of IORT-wound fluid on breast tumors containing all cell types. The current study is the first at studying the effects of IORT-wound fluid on proliferation, apoptosis, and EMT in human-derived tumors in microfluidic devices, providing interaction of immune, non-immune, and cancer cells. In natural circumstances during the surgery and IORT therapy, residual tumor cells in the negative tumor bed were exposed to irradiation. Nevertheless, here we prepared the spheroids from non-irradiated tumor tissues. So, it is not fair to compare the effects of WFs on irradiated residual tumor cells in the body with the non-irradiated tumor spheroids in the device, and here could just study the bystander effect. We suggest more experiments based on similar circumstances to tumor cells and the tumor microenvironment.

## Methods

All the methods were performed in accordance with the ethics committee guidelines.

### Patients

Patients were recruited among women with breast cancer who were referred to the Cancer Research Center of Shahid Beheshti University of Medical Sciences (Tehran, Iran) between 08/2018 to 11/2019 based on the Guideline criteria of IORT: age between 28 and 74 years and no previous history of radiotherapy, chemotherapy or surgery associated with the ongoing disease. This clinical trial was approved by the University Ethics Committee (Code NO. IR.SBMU.CRC.1398.021). Patients were subdivided into two groups, including the “WF-RT” group, women who underwent IORT (X-ray, boost dose, 20 Gy, 1 h) (n = 10), and the “WF” group, women who only underwent conservative surgery (n = 10). Patients' information, including age, marital status, tumor grade, HER2, ER, PR, and Ki67 status, is presented in Supplementary Table S1. According to reports from the pathology lab, all the tumor samples were invasive ductal carcinoma. The patients in the WF group were matched with the WF-RT group. Written informed consent was obtained from individual patients, and the Ethics Committee approved the experimental protocol of Shahid Beheshti University of Medical Sciences (Code NO. IR.SBMU.CRC.1398.021).

### Tissue and surgical wound fluid samples

Twenty freshly collected samples from stage II/III tumors were obtained during surgery. One section from each sample was sent to the pathology laboratory, while the remaining tissue was delivered to the cell culture laboratory within 30 min after the tumor resection. Drainage wound fluid (the formed fluid induced by surgery in the tumor cavity after the removing as we name in this study, “WF”) collected over the first 24 h after lumpectomy were obtained from each patient^13,15,91,92^. All WFs were centrifuged and filtered under sterile conditions and stored at − 80 °C. In the following sections of this study, the term WF indicates wound fluid from patients who only went under surgery(control group), and the term WF-RT indicates wound fluid from patients who received IORT during the surgery(test group).

### Cell lines, 2D cell culture, and proliferation assay

Three human breast cancer cell lines, including MCF-7 (ER-positive, PR positive, HER2 negative), MDA-MB-231 (ER-negative, PR negative, HER2 negative), SKBR3(ER-negative, PR negative, HER2 positive), and a non-tumorigenic epithelial cell line, MCF10, obtained from Iranian Biological Research Centre, were cultured in Dulbecco’s Modified Eagle’s Medium (DMEM) supplemented with 10%FBS, 1%pen/strep and 2 mM l-glutamine. The MCF-7 cells received more supplementary materials (DMEM/F12 medium with EGF, hydrocortisone, cholera toxin, and insulin). Cells were grown at 37 °C in a humidified atmosphere with 5% CO_2_. All the cells were treated with 10%WF/WF-RT in DMEM without FBS, and the MTT assay was then performed to investigate cell viability under WF treatment. Briefly, cells were seeded onto 96-well culture plates at a density of 1 × 10^4^ cells/well and were treated with all individual samples (WF/WF-RT) at different concentrations. The cell viability was measured at an absorbance of 570 nm using an ELISA reader. The absorbance values were measured as percentages of controls(CTR), yielding percentage cell viability after 24, 48, and 72 h of treatment with WFs.

### Cell cycle assay by flow cytometry

The effect of WF/WF-RT compounds on cell cycle distribution was examined using flow cytometry. 4 × 10^5^ cells were treated with 10% of WF/WF-RT in DMEM cell culture medium for 48 h. Then, the cells were collected by centrifuging at 2000 rpm for 5 min. The pellets were fixed by cold ethanol. Then, PBS-EDTA-BSA was added before centrifugation (2000 rpm for 5 min). Afterward, the washing buffer, included EDTA (20 mg), PBS (100 mL), BSA (1 g), and sodium azide (100 mg), was utilized to wash the pellets before adding a staining buffer that contained PBS (1 mL), PI (0.3 μg/mL), Rnase (50 μg/mL), Triton X-100 (1 μL/mL) and EDTA (0.37 mg/mL). A flow cytometer was employed to analyze the cells after 30 min of incubation on ice. The percentage of the cells in the G1, S, and G2 phases were analyzed by Flowjo 7.6.

### Apoptosis assay by flow cytometry

Apoptotic effects of WF/WF-RT compounds against MDA-MB-231 cells were examined using an Annexin V FITC Apoptosis Detection Kit I (bioscience annexin v apoptosis detection kit FITC, USA). 6 × 10^5^ cells were treated with 10%WF/WF-RT in DMEM for 48 h. Then, the cells were collected by centrifuging (2000 rpm for 5 min). The pellets were washed in 100 μL of binding buffer. Afterward, cells were incubated on ice in the dark for 15 min with a mixture of 5 μL PI and 5 μL AnnexinV. Finally, 400 μL of binding buffer was loaded, and the analysis by employing a BD FACSCalibur flow cytometer (Becton Dickinson, USA). Untreated cells were considered a negative control. Data from 10 000 cells were collected in each data file. Four different populations of cells were easily distinguished: 1—unlabelled (viable cells), 2—bound Annexin VFITC only (early apoptotic), 3—stained with PI (necrotic), and 4—both bound Annexin V-FITC and been labeled with PI (late apoptotic/necrotic cells). The fluorescence population was revealed as a two-color dot plot analysis, and the fluorescent cells % in each quadrant was established.

### Clonal survival assay

The MCF-7 cells were selected for showing colony formation (colony abundance and shape; holoclone, meroclone, and paraclone). The terms holoclone, meroclone, and paraclone have since become synonymous with colonies derived from the stem, early, and late-stage transit-amplifying cells, respectively. Holoclones and meroclones comprise highly proliferative, immortal cells that can self-renew and serially tumorigenic but differ in proportions. Meroclones contain a smaller proportion of self-renewing stem cells than holoclones. Both holoclones and meroclones carried immortal cells, which, when serially cloned, could be cultured for more than 100 divisions, whereas paraclones were terminal. Meroclones had a longer latency than holoclones and developed smaller tumors, again hinting that meroclones comprise fewer stem cells than holoclones^32^. After WF/WF-RT treatment (10% in DMEM) for 48 h, the cells were trypsinized and suspended in fresh medium (DMEM with 10% FBS), quantified, and 500 to 1000 cells were plated in triplicate into 24-well cell culture plates. After 7 days, colonies were fixed with paraformaldehyde and stained with crystal violet as previously described. The number of colonies and their shapes in each plate were determined by phase-contrast microscopy. Data were presented as mean colony number ± SD relative to untreated controls (n = 3 independent experiments). In this assay, we used both individual patient samples (WF from the control group and WF-RT from the test group) and the pool of all WFs in every group.

### In vitro scratch-induced migration assay

A scratch wound test was administered to evaluate the motility of MDA-MB-231cells. The MDA-MB-231 cells (7 × 10^5^ cells/well of 24-well plates) were treated with the WF/WF-RT (individual and pooled samples) in variable concentrations (10% of pooled samples) for 48 h. The cells were incubated with mitomycin (0.5 mg/mL) for two h, then were wounded by the tip of a sterile 200-μL micropipette, washed by PBS three times to remove all separated cells. Then the medium was replaced with DMEM containing 2% FBS for both WFs-treated and control groups. Images were taken by a microscope (Nikon, ECLIPSE, TE 2000-4, Japan) at 0 and 24 h to detect the migration rate. ImageJ macros of scratch analysis quantified the migrated cells. The scratch line in 24 h mines from 0 h and data were presented as mean scratch line ± SD relative to untreated controls (n = 3 independent experiments).

### Spheroid preparation from breast tumor samples and microfluidic cell culture

According to the described protocol for tumor spheroid preparation, tissue specimens were received in RPMI on ice and processed in a sterile dish^21,22^. Samples were processed mechanically and enzymatically using scalpel, forceps, and collagenase type I. processed specimens were suspended in RPMI 1640 with 10% FBS and subsequently filtered to obtain the proper fractions (40-100 µm). Cell pellet containing 40–100 µm spheroids was then resuspended in collagen hydrogels (type I rat tail collagen 2.5 mg/ml, Corning Co.). Hydrogels containing spheroids were injected into the device's central channels and incubated for 30 min at 37 °C in humidified chambers. Microfluidic chips were designed and manufactured at AIM BIOTECH (DAX-1, AIM BIOTECH, https://www.aimbiotech.com)^20–22^. We used three devices for each sample (as three replications for the experiments). Following incubation, hydrogels were hydrated with RPMI + 10% FBS and incubated at 37 °C for 24 h. On days 1, 2, 3, 4, and 5, spheroids were treated with WF and evaluated microscopically every 24 h for 6 days.

### Live/dead staining of tumor spheroids

Spheroid viability was evaluated after staining with AO/PI (Nexcelom ViaStain AO/PI staining solution (Nexcelom, CS2-0106) according to the manufacturer protocol on day 6^19^. Besides, to assess prepared spheroids viability before injection to the devices, we used AO/PI staining.

### Immunofluorescence staining for cell lines and tumor-derived spheroids

The MDA-MB-231 cells were treated with two groups of WF (10% of pooled samples in RPMI without FBS) for 48 h, and hydrogels containing spheroids (in central channels of the devices in both WF and WF-RT groups) were washed with PBS, fixed using 4% paraformaldehyde solution for 10 min, and then permeabilized with 0.3% Triton for 30 min to evaluate the migration and apoptosis. MDA-MB-231 cells were fixed and treated with primary antibodies of P16 (sc1661) and P21(sc6246) for senescence detection and MMP-9 (sc13520) for evaluation of invasion. Both cell lines and spheroids were treated with primary antibodies of Caspase3(ab4051) to evaluate apoptosis and E-cadherin (ab1416) for evaluation of invasion. After an overnight treatment, they were incubated with the secondary anti-mouse (FITC:sc-2010) for green color and secondary anti-mouse (PE: sc-3738) for red color. Antibodies were diluted 1:150 in PBS and used according to the manufacturer's protocol. A selection of three random fields was photographed and counted by fluorescence microscopy.

### Imaging of spheroids

For live/dead imaging, spheroids were imaged on an inverted Nikon Eclipse Ti microscope equipped with a Nikon DS-Qi1Mc camera using NIS-Elements software. The total area of Acridine orange-stained live (green) cells vs. Propidium iodide-stained dead (red) cells was quantified. Also, in fluorescence imaging, the total area of Caspase 3 expressing (green) cells and E-cadherin expressing (red) cells was quantified using the method described above.

### Validation of protein expression by GEPIA database and ITRAQ result

Gene expression profiling interactive analysis (GEPIA) database (http://gepia.cancer-pku.cn/) was used to identify differential expression of P16, P21, casp3, E-cad, and MMP9 (shown graphically) between normal tissue and breast cancer tissue and the association between the expression of E-cad and tumor stages. GEPIA (http://gepia.cancer-pku.cn/) is a web tool that presents fast and customizable functionalities based on data retrieved from The Cancer Genome Atlas (TCGA; https://tcga-data.nci.nih.gov/tcga/). Differential analysis was performed using one-way ANOVA, using disease state or tumor stage as the variables for assessing differential expression^93^. Selected BRCA datasets matched with TCGA normal data, and Log2FC Cutoff was selected 1, and the p-value Cutoff 0.01. In Our previous study^14^, we used the iTRAQ (Isobaric tag for relative and absolute quantitation) technique to investigate proteome profile of tumor bed tissue samples that had been collected from patients treated with intraoperative electron radiotherapy (IOeRT), 21 Gy (collected sample before and 24 h of post-treatment with IOeRT). By iTRAQ for proteome quantification, in total, 1,045,410 spectrums were generated; likewise, 5860 proteins were identified (FDR < 0.01). We searched the genes P16, P21, Casp3, E-cad, and MMP9 in proteome profiles to find protein expression in tumor bed tissue after treatment with irradiated WF. Tumor tissue samples were also analyzed by the ITRAQ technique to find the protein profile compared to their margins (unpublished data) to study tumor bed tissue. The data were represented as Mean-Ratio and Q-value < 0.05. The Q-value estimates the expected positive false discovery rate obtained by rejecting the null hypothesis for any result with an equal or smaller Q-value. We also used this data to find the differential protein expression of tumor tissue compared with normal margin tissue. We applied DAVID Bioinformatics Resources 6.8 (https://david.ncifcrf.gov/)^94^ to identify the KEGG pathway^27^ enrichment.

### Statistical analysis

The GraphPad Prism software program (v.6) was used to conduct statistical analyses (GraphPad Software, Inc., La Jolla, CA, USA). A t-test was used to analyze the data. A statistically significant difference was described as a P-value and Q-value of less than 0.05.

### Ethical approval

Written informed consent was obtained from individual patients, and the Ethics Committee approved the experimental protocol of Shahid Beheshti University of Medical Sciences (Code NO. IR.SBMU.CRC.1398.021).

## Supplementary Information

Supplementary Information.

Supplementary Tables.
