# Supplementary material for: Radiobiological effects of wound fluid on breast cancer cell lines and human-derived tumor spheroids in 2D and microfluidic culture
Source: Sci Rep. 2022 May 10;12:7668. doi: 10.1038/s41598-022-11023-z (PMC9091274; doi:10.1038/s41598-022-11023-z)
Supplement: Supplementary file 1 — Supplementary Information. [file 41598_2022_11023_MOESM1_ESM.docx]

Supplementary data:

**Radiobiological effects of wound fluid on breast cancer cell lines and**

**human-derived tumor spheroids in 2D and microfluidic culture**

Shabnam Jeibouei^1,2,#^, Ali Hojat^1,#^, Ebrahim Mostafavi^3,4^, Amir Reza Aref^5,6^, Alireza Kalbasi^5^, vahid Niazi^7^, Mohammad Ajoudanian^7^, Farzaneh Mohammadi^8^, Fariba Saadati^9^, Seyed Mohammadreza Javadi^10^, Forough Shams^2^, Maryam Moghaddam^11^, Farshid Karami^7^, Kazem Sharifi^2^, Farid Moradian^12^, Mohammad Esmaeil Akbari*^1^, Hakimeh Zali**^7^

1. Cancer Research Center, Shahid Beheshti University of Medical Sciences, Tehran, Iran

2. Department of Medical Biotechnology, School of Advanced Technologies in Medicine, Shahid Beheshti University of Medical Sciences, Tehran, Iran

3. Stanford Cardiovascular Institute, Stanford University, Stanford, CA, USA

4. Department of Medicine, Stanford University School of Medicine, Stanford, CA, USA

5. Brigham and Women’s Hospital, Harvard Medical School, Boston, MA, USA

6. Belfer Center for Applied Cancer Science, Dana-Farber Cancer Institute, Boston, MA, USA

7. Department of Tissue Engineering and Applied Cell Sciences, School of Advanced Technologies in Medicine, Shahid Beheshti University of Medical Sciences, Tehran, Iran

8. Department of Biology, Central Tehran Branch, Islamic Azad University, Tehran, Iran

9. ZIK*plasmatis*, Leibniz Institute for Plasma Science and Technology (INP), Greifswald, Germany

10. Department of Surgery, School of Medicine, Besat Hospital, Hamadan University of Medical Sciences, Hamadan, Iran

11. Department of Molecular and Cell Biology, Faculty of Life Sciences and Biotechnology, Shahid Beheshti University, Tehran, Iran

12. Shohadaye Tajrish Hospital, Shahid Beheshti University of Medical Sciences, Tehran, Iran

# These authors contributed equally to the manuscript

*Corresponding author: Mohammad Esmaeil Akbari, Cancer Research Center, Shahid Beheshti University of Medical Sciences, Tehran, Iran

E-mail: [profmeakbari@gmail.com](mailto:profmeakbari@gmail.com)

Tel: +989120193314

 **Co-corresponding author: Hakimeh Zali, Department of Tissue Engineering and Applied Sciences, School of Advanced Technologies in Medicine, Shahid Beheshti University of Medical Sciences, Tehran, Iran

E-mail: hakimehzali@gmail.com, [h.zali@sbmu.ac.ir](mailto:h.zali@sbmu.ac.ir)

**Supplementary Table S1**. **Patients’ information.** The table represents data including age, marital status, HER2, ER, PR, and Ki67 status and hystopatologic type of the tumor related to 20 breast cancer patients who participated in this study.

| **Sample’s number** | **Date of surgery** | **Age** | **Marital status** | **IORT** | **Pathology result** | **HER2, ER, PR, Ki67** | **Tumor grade** |
| --- | --- | --- | --- | --- | --- | --- | --- |
| **S1** | 28/08/2018 | 39 | Married | - | Invasive ductal carcinoma | + - - 20% | II |
| **S2** | 4/09/2018 | 50 | Married | - | Invasive ductal carcinoma | - - - 60% | II |
| **S3** | 9/09/2018 | 72 | Married | + | Invasive ductal carcinoma | - + + 60% | III |
| **S4** | 11/09/2018 | 49 | Married | + | Invasive ductal carcinoma | - + + 10% | II |
| **S5** | 16/09/2018 | 74 | Married | - | Invasive ductal carcinoma | - + + 70% | III |
| **S6** | 23/09/2018 | 33 | Married | + | Invasive ductal carcinoma | + + + 30% | III |
| **S7** | 25/09/2018 | 43 | Married | + | Invasive ductal carcinoma | + + + 5% | III |
| **S8** | 30/09/2018 | 29 | Married | - | Invasive ductal carcinoma | - + - 10% | II |
| **S9** | 2/10/2018 | 57 | Married | + | Invasive ductal carcinoma | - + + 20% | II |
| **S10** | 9/10/2018 | 47 | Married | + | Invasive ductal carcinoma | - + + 10% | III |
| **S11** | 14/10/2018 | 49 | Married | + | Invasive ductal carcinoma | + - - 5% | III |
| **S12** | 14/10/2018 | 28 | Single | + | Invasive ductal carcinoma | + + + 20% | II |
| **S13** | 16/10/2018 | 69 | Married | + | Invasive ductal carcinoma | - + + 60% | III |
| **S14** | 29/10/2018 | 40 | Married | + | Invasive ductal carcinoma | + + + 5% | II |
| **S15** | 9/03/2019 | 60 | Married | - | Invasive ductal carcinoma | - + + 30% | II |
| **S16** | 2/10/2019 | 45 | Married | - | Invasive ductal carcinoma | - + + 15% | III |
| **S17** | 3/11/2019 | 38 | Married | - | Invasive ductal carcinoma | + + + 10% | II |
| **S18** | 5/11/2019 | 50 | Married | - | Invasive ductal carcinoma | + - - 5% | III |
| **S19** | 5/11/2019 | 50 | Married | - | Invasive ductal carcinoma | - + + 5% | III |
| **S20** | 5/11/2019 | 37 | Married | - | Invasive ductal carcinoma | - + + 15% | III |

100µm

**A)**

**
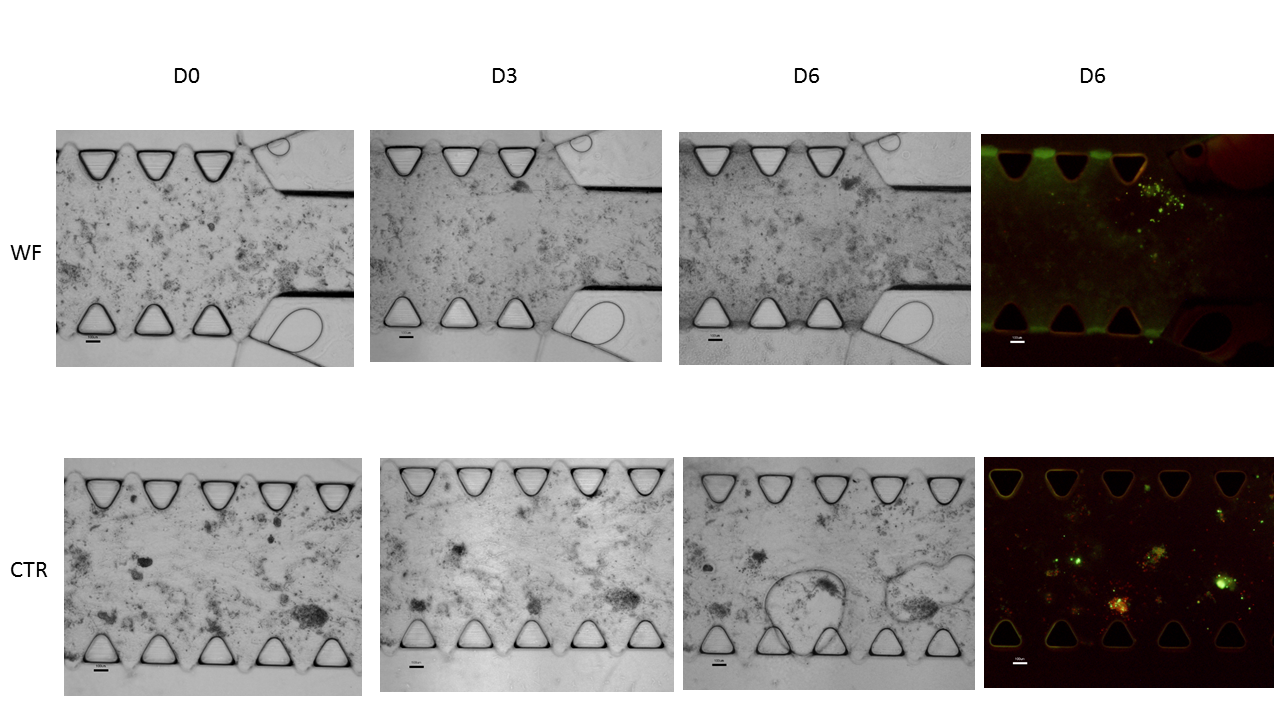
**

**
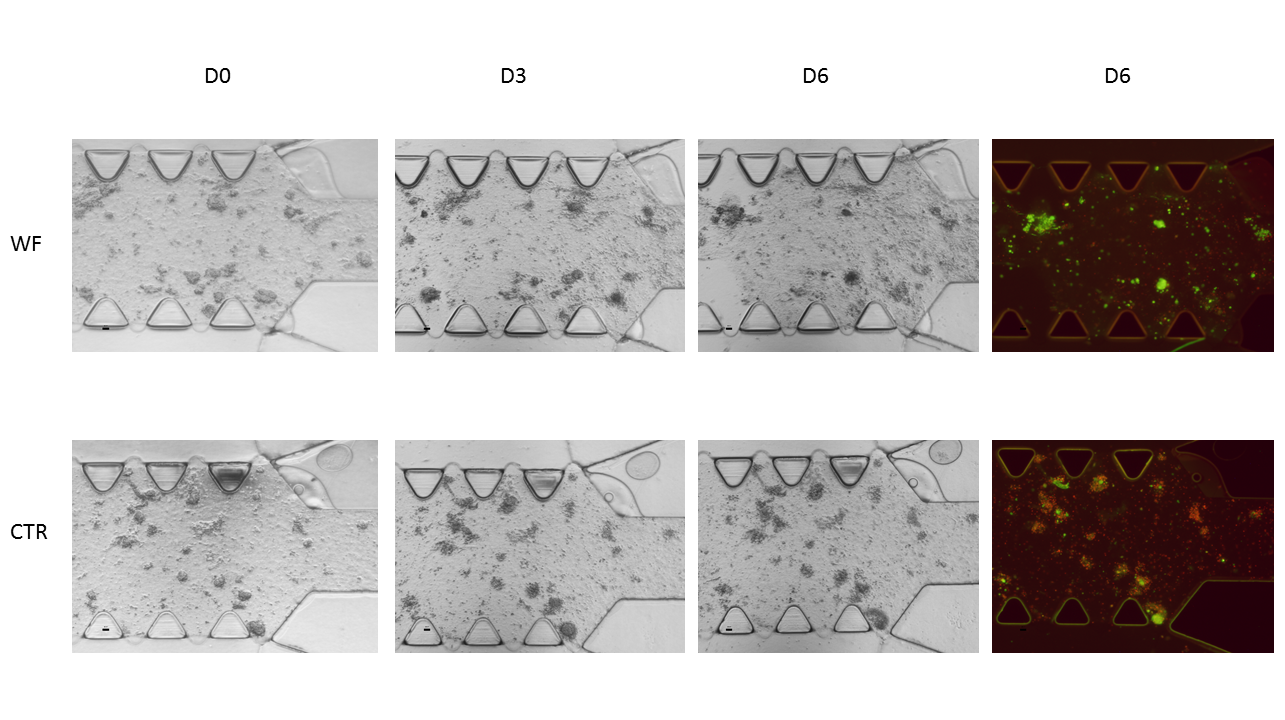
B)**

**B)**

**Supplementary Figure S1. Optical and fluorescent images of spheroids on day 0 to day 6. A)**The WF treated tumor-derived spheroids. Scale bars: 100µm, Original magnification: 40x. **B)** The WF-RT-treated spheroids. Scale bars: 40µm, Original magnification: 40x. In both figures **A** and **B**, the images from treated spheroids with RPMI+10% FBS introduce CTR. Inverted phase-contrast microscopy and fluorescent microscopy. Original magnification: 40x. Green: AO/live cells; Red: PI/dead cells. Scale Bar: 40µm.

**A)**


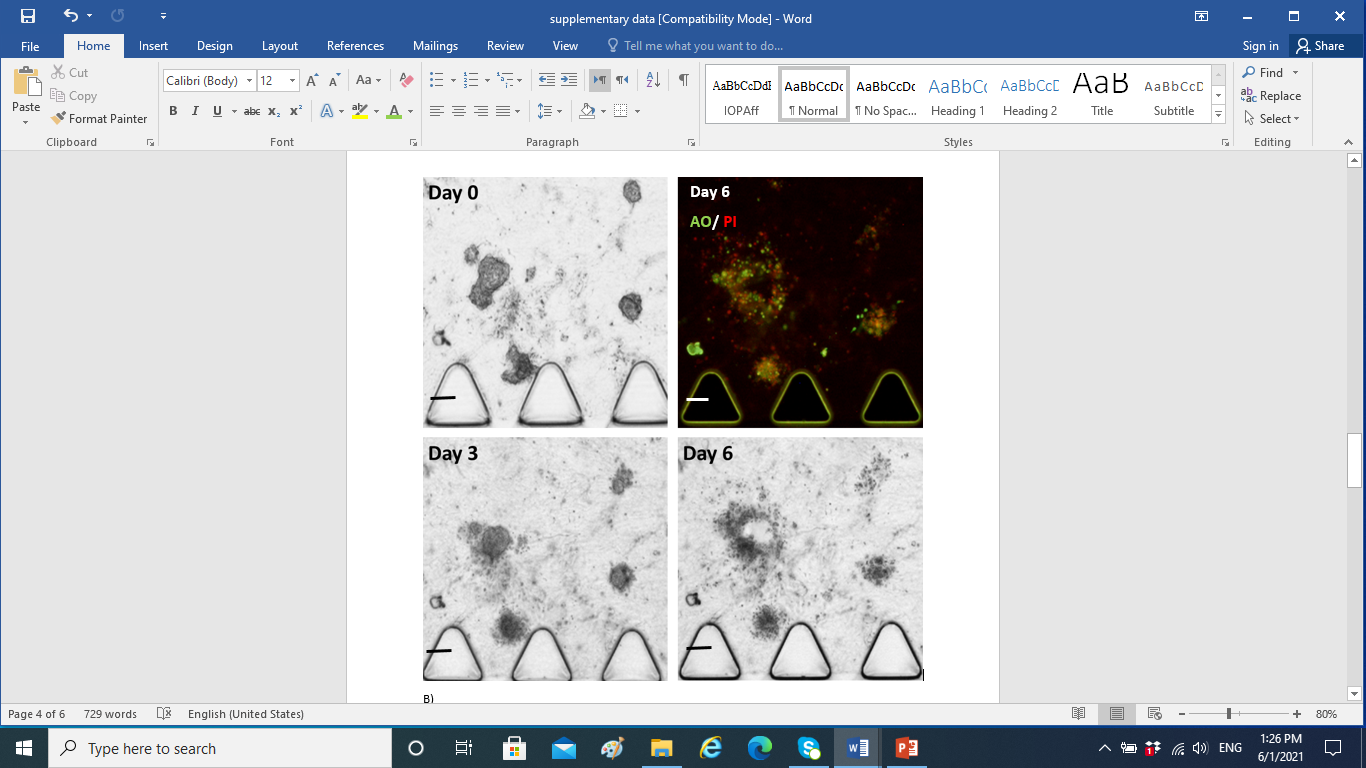


**B)**


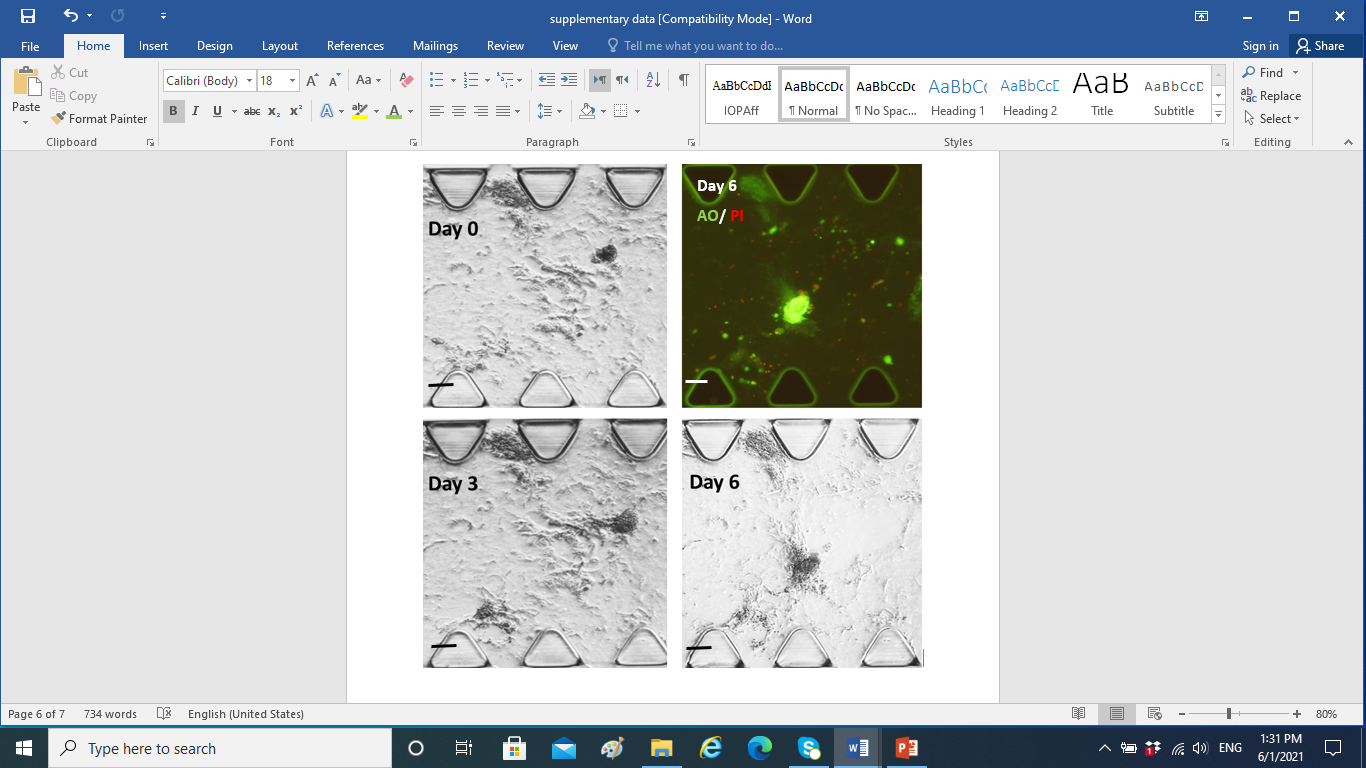


**Supplementary Figure S2.** Optical and fluorescent images of CTR spheroids migration in days0, 3, and 6. **A)** the RPMI-treated tumor spheroids derived from pateints underwent surgery only and **B)** the RPMI-treated spheroid sample from pateints recived surgery plus IORT. Inverted phase-contrast microscopy and fluorescent microscopy. Original magnification: 40x. Green: AO/live cells; Red: PI/dead cells. Scale Basr: 100µm.

**Day 6**
